# Light-activated photocurrent degradation and self-healing in perovskite solar cells

**DOI:** 10.1038/ncomms11574

**Published:** 2016-05-16

**Authors:** Wanyi Nie, Jean-Christophe Blancon, Amanda J. Neukirch, Kannatassen Appavoo, Hsinhan Tsai, Manish Chhowalla, Muhammad A. Alam, Matthew Y. Sfeir, Claudine Katan, Jacky Even, Sergei Tretiak, Jared J. Crochet, Gautam Gupta, Aditya D. Mohite

**Affiliations:** 1 Materials Physics and Application Division, Los Alamos National Laboratory, Los Alamos, New Mexico 87545, USA; 2 Chemistry Division, Los Alamos National Laboratory, Los Alamos, New Mexico 87545, USA; 3 Theoretical Division, Los Alamos National Laboratory, Los Alamos, New Mexico 87545, USA; 4 Center for Functional Nanomaterials, Brookhaven National Laboratory, Upton, New York 11973, USA; 5 Department of Materials Science and Engineering, Rutgers University, 607 Taylor Road, Piscataway, New Jersey 08854, USA; 6 School of Electrical and Computer Engineering, Purdue University, West Lafayette, Indiana 47907, USA; 7 Institut des Sciences Chimiques de Rennes, ISCR UMR 6226, CNRS, Université de Rennes 1, 35042 Rennes, France; 8 Fonctions Optiques pour les Technologies de l'Information, FOTON UMR 6082, CNRS, INSA de Rennes, 35708 Rennes, France

## Abstract

Solution-processed organometallic perovskite solar cells have emerged as one of the most promising thin-film photovoltaic technology. However, a key challenge is their lack of stability over prolonged solar irradiation. Few studies have investigated the effect of light soaking on hybrid perovskites and have attributed the degradation in the optoelectronic properties to photochemical or field-assisted ion migration. Here we show that the slow photocurrent degradation in thin-film photovoltaic devices is due to the formation of light-activated meta-stable deep-level trap states. However, the devices can self-heal completely by resting them in the dark for <1 min or the degradation can be completely prevented by operating the devices at 0 °C. We investigate several physical mechanisms to explain the microscopic origin for the formation of these trap states, among which the creation of small polaronic states involving localized cooperative lattice strain and molecular orientations emerges as a credible microscopic mechanism requiring further detailed studies.

## Abstract (web summary)

Organometallic perovskite solar cells exhibit good efficiency but their photostability is still relatively poorly understood and controlled. Here the authors show that photo-degradation arises from the formation of light-activated meta-stable trap states, is reversible, and can be frozen at 0 °C.

## Graphical abstract

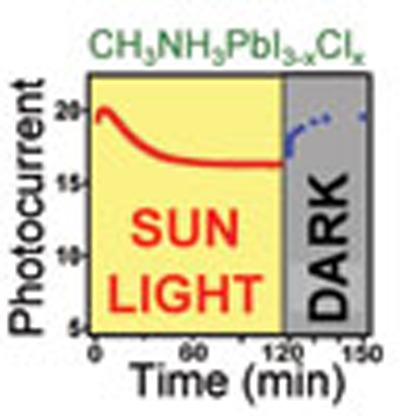

Organometallic halide perovskite solar cells are promising because of their high power conversion efficiency (PCE) up to 15–20% achieved for methyl ammonium lead iodine (MAPbI_3_, MA=CH_3_NH_3_) materials^1^,^2^,^3^,^4^. While achieving high PCE is important, the successful translation of proof-of-concept devices into low-cost high efficiency photovoltaic technology requires stable operation under solar illumination and in air. The problem of stability against moisture can be circumvented through encapsulation schemes,^5^,^6^,^7^,^8^,^9^,^10^ but photo-stability of perovskite devices remains an open question^11^,^12^. There have been just a handful of reports on understanding the photo-stability in perovskite solar cell devices have attributed the degradation in the device performances to either ultraviolet-assisted damage to the active layer^13^,^14^,^15^,^16^ or ion migration^13^,^16^,^17^,^18^,^19^,^20^,^21^,^22^,^23^. Such degradation over time with solar illumination has the potential to seriously undermine the commercialization of perovskite-based solar cells.

Here we demonstrate that the degradation of hybrid perovskite solar cell performance with constant solar illumination in crystalline large-grain-size perovskite solar cells is due to the degradation of the photocurrent, which, importantly, can rapidly (<1 min) recover (self-heal) in the dark to its original value. Capacitance measurements on devices and time-resolved photoluminescence (PL) spectroscopy on thin films attribute the observed photocurrent degradation to the formation of light-activated meta-stable trap states, which over prolonged illumination (light soaking) leads to the accumulation of space charges. Near-infrared absorption and low-frequency dielectric constant directly probe these light-activated meta-stable trap states after light soaking as mid-gap states. Using extensive device characterization and optical spectroscopy measurements, we evaluate the possible role of intrinsic traps, ion migration, ferroelectric effect or small polaronic states as physical origins for the formation of these light-activated trap states. Our overall analysis of the photo-degradation process, including both experimental data and density functional theory (DFT), suggests accumulation of these light-activated meta-stable trap states into the formation of macroscopic charged regions can result in non-homogeneous electric fields in the perovskite layer resulting in slow degradation of the photocurrent. We further demonstrate that the PCE degradation can be dramatically slowed down by simply lowering the device temperature from 25 to 0 °C. From a practical perspective, the observed fast self-healing (<1-min at 70 °C) or stabilization of the PCE is an important step towards the realization of photo-stable perovskite solar cells.

## Results

### Device characterization with constant light illumination

**Figure 1:**
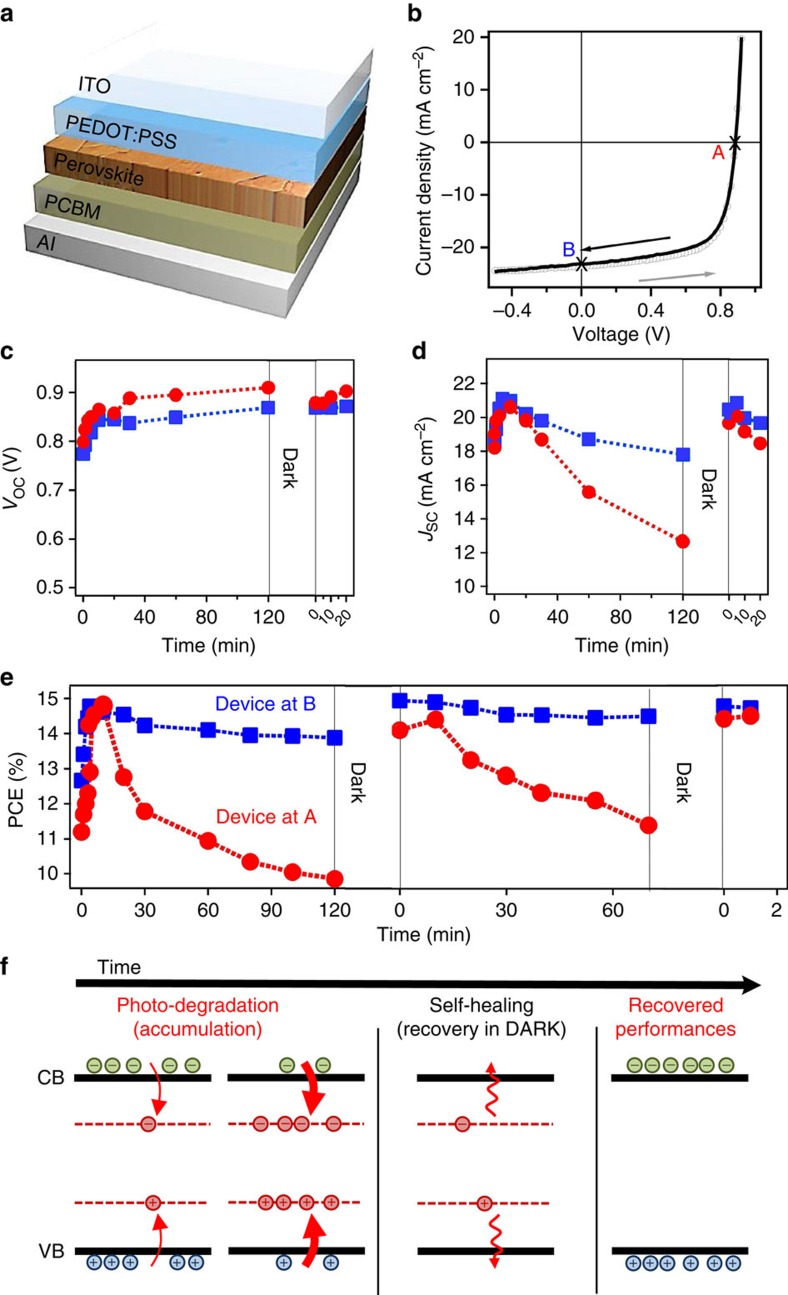
Solar cell performance under constant illumination. (**a**) Planar solar cell architecture used in this study. (**b**) Typical device current density-voltage (*J–V*) curves under 1-Sun illumination showing no hysteresis for crystalline large-grain-size organometallic perovskite used in this study. (**c**–**e**) Time evolution of device performance and figures of merit under constant 1-sun illumination and after resting the device in dark. Open-circuit voltage c, short-circuit current density d, and power conversion efficiency e, when the device is stressed at point A (red circles, *J*=0, *V*=*V*_OC_) or at point B (blue squares, *J*=*J*_SC_, *V*=0) between each data point. (**f**) Schematics of the proposed photocurrent degradation and self-healing mechanism based on perovskite layer band structure evolution sketching the valence (VB) and conduction (CB) bands for three situations: during photo-degradation and accumulation, during recovery in dark and under illumination after self-healing. The red dotted lines refer to light-activated meta-stable trap states that relax in dark returning the device to its steady state. Arrows sketch how photo-generated carriers can populate those light-activated trap states under light or relax in dark over time.

Figure 1b shows a typical *J–V* measurement under 1-Sun illumination ((Air Mass (A.M.) 1.5) with no hysteresis using the device structure illustrated in Fig. 1a, consistent with our previous measurements^19^. The time evolution of the solar cell figure of merits when the device is stressed at open-circuit (point A in Fig. 1b) or short-circuit (point B in Fig. 1b) condition are illustrated in Fig. 1c–e (see also Supplementary Fig. 1). Initially an increase in short-circuit current (*J*_SC_), open-circuit voltage (*V*_OC_) and PCE are observed after light soaking the device for ∼10–20 min under 1-Sun illumination before reaching steady state. Our study focuses on device performance after steady-state operation has been achieved. Once the *V*_OC_ reaches its steady-state value, it does not degrade over time, independent of the stress condition. On the other hand, *J*_SC_ decreases by ∼40% after 2 h of constant 1-Sun illumination when the device is stressed at an open-circuit condition (point A). In contrast, for the device stressed at short-circuit condition (point B), the *J*_SC_ decreases by merely 10% in 2 h. In both cases, since the *V*_OC_ remains unchanged, the time evolution of PCE (Fig. 1e) follows the trend established by the *J*_SC_. Figure 1c–e shows that the device parameters can self-heal to their original steady-state values after resting the device in the dark.

These observations were confirmed independently on several devices from several batches (summarized in Supplementary Fig. 2 and Supplementary Note 1).

We excluded any noticeable light-activated chemical decomposition by verifying the stability under solar cell operation of X-ray diffraction (XRD) Bragg peak pattern, photo-emission properties and photocurrent spectrum (Supplementary Figs 3–4 and Supplementary Note 2). Additional XRD spectrums are attached in Supplementary Fig. 2 for different films. In addition, we verified that device heating observed at steady-state operation, forward bias, and perovskite-crystal structure does not influence the intrinsic mechanism of photo-degradation reported in this study (Supplementary Figs 5–7 and Supplementary Note 3). Furthermore, the photocurrent degradation and recovery were studied in more detail with different types of techniques (for example, *C*–*V* measurements and photocurrent transient) and also under different operation conditions of external bias, light-excitation intensity and operation temperature (see details in Supplementary Figs 8–12 and Supplementary Note 4). Some of these results will be discussed later. All these measurements suggest that the photocurrent degradation in solar cells is truly induced by light soaking.

To understand the observations in Fig. 1c–e, we propose a mechanism based on changes in the sub-gap density of states over illumination time in the perovskite active layer as illustrated in Fig. 1f. Under constant 1-Sun illumination, there is an increasing number of light-activated meta-stable states (dashed red lines) that accumulate over tens of minutes to hours leading to the formation of charged regions in the bulk of the thin film, which, in turn, results in the degradation of the photocurrent (photo-degradation regime). However, when the device is rested in the dark, the majority of these light-activated trap states dissipate away, and on re-illumination the photocurrent (and hence PCE) self-heals to nearly 100% of its initial steady-state value (self-healing/recovery regime).

### Characterization of light-activated meta-stable trap states

**Figure 2:**
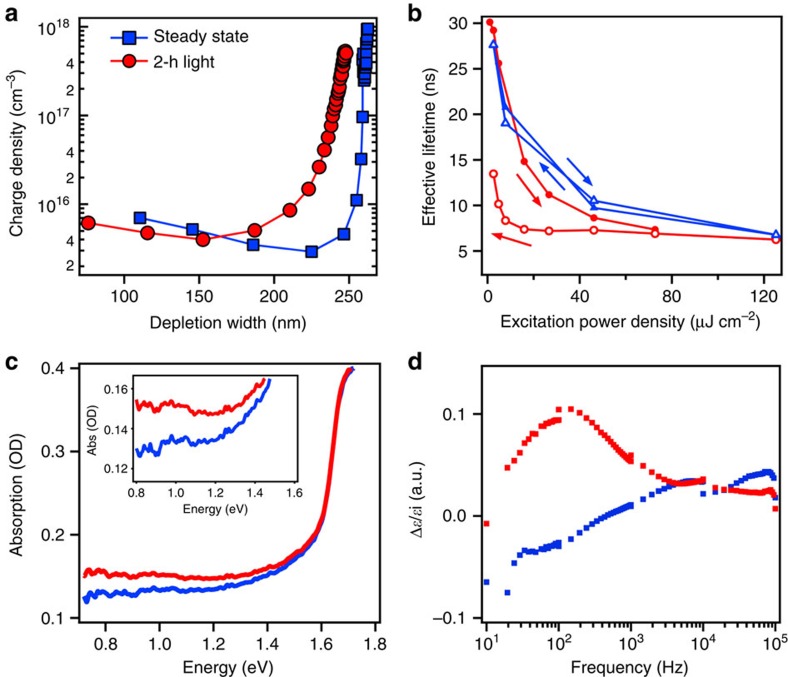
Evidence and characterization of light-activated meta-stable trap states. (**a**) Charge density profile of the device steady state and after illumination for 2 h at an open-circuit condition. The right-hand side of the *x*-axis corresponds to the interface perovskite/contact layers, and the left-hand side locates the center of the active perovskite layer. (**b**) Light-excitation power density dependence of the effective PL lifetime of a thin film under continuous light soaking (red) and after recovery of the system in the dark between each data point (blue). Filled and open symbols represent excitation power density increase and decrease cycle, respectively. Lines between symbols are a guide to the eye. (**c**) Direct evidence into the deep-level trap states formed after white light soaking (red line) as compared with steady state thin (blue line) by near-infrared absorption measurement. The inset is zoomed-in region. (**d**) Low-frequency real dielectric constant relative change (Δ*ɛ*/*ɛ*_i_) for a device illuminated for 2 h (red) and after recovery in dark (blue), as compared with steady state.

The presence of the light-activated trapped charges was detected through several independent measurements. Figure 2a illustrates the charge density profile measured using capacitance–voltage measurements (for details see Supplementary Fig. 10). The space charge density increases from ∼5 × 10^15^ cm^−3^ before photocurrent degradation (blue) to ∼10^16^ cm^−3^ after a 2-h illumination under an open-circuit condition (red) indicating the presence of trapped charges near the depletion region edge (∼200 nm along the film thickness). Evidence of trapped charges is also corroborated using photocurrent transient measurements, which show that the photocurrent discharging time constant (when the device is switched from open-circuit to short-circuit under light) increases with constant illumination over time (Supplementary Fig. 11). Furthermore, distinct signatures of these light-activated trap states were observed in the power-dependent PL spectral and temporal responses by comparing the response of a perovskite thin film under continuous light soaking and the one relaxed in dark between each power density measurement point (Supplementary Figs 13–14 and Supplementary Note 5). In the former case, a decrease in the effective recombination lifetime (Fig. 2b) and PL intensity (Supplementary Figs 15 and 16) was observed. More importantly, the detected signal exhibits strong hysteresis when sweeping laser power from low to high and back to low again. We attribute these effects in the PL to an increase in the non-radiative recombination via light activation of meta-stable trap states and to photo-charging effects, both triggered either over time or at high excitation power density as reported in devices (see details in Supplementary Note 5). These detrimental effects are not observed when the film response is measured after resting the sample in the dark, which allows for the relaxation of majority of these light-activated trap states.

Additional characteristics of the light-activated meta-stable trap states were directly probed by near-infrared absorption spectroscopy (Fig. 2c) and impedance spectroscopy examining light-induced change of the material dielectric constant (Fig. 2d). Figure 2c illustrates the near-infrared thin-film absorption before and after white light soaking, which shows a clear increase in the near-infrared absorption extending >500 meV deep within the band gap. Moreover, we also probed the presence of these light-activated mid-gap states using impedance spectroscopy (Fig. 2d and Supplementary Fig. 17). By measuring the device dielectric constant as a function of alternating current (AC) field frequency before and after illumination, the low frequency (from 10 to 10^3^ Hz) dielectric constant exhibits a clear increase after 2 h of illumination relative to the steady state (Fig. 2d, red). We emphasize that the fact that the dielectric constant can relax in the dark (Fig. 2d, blue) clearly suggests that it strongly correlates with the observed photocurrent degradation and self-healing behaviour.

### Recovery dynamics of photocurrent and PL

**Figure 3:**
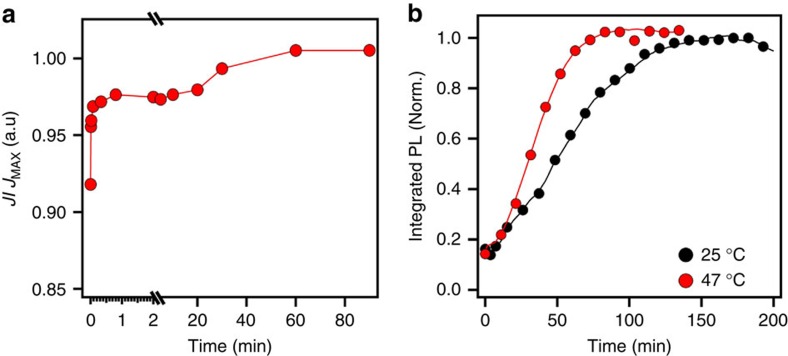
Investigation of the recovery process of perovskite device and thin film. (**a**) photocurrent and (**b**) photoluminescence probed recovery dynamics under light while otherwise resting the device or perovskite film in the dark. The lines are a guide to the eye.

The recovery dynamics of *J*_SC_ were also investigated by probing the device with 1-Sun illumination after photo-degradation, while otherwise resting it in dark between measurements (Fig. 3a). We observe two distinct recovery time regimes: a fast recovery (<1 min), which brings the device to about 96% of its steady-state value, followed by a longer recovery time of up to tens of minutes to an hour to fully restore the device photocurrent. We attribute the fast component to the relaxation of the majority of the light-activated trap states in dark. The slow component is most likely due to the reminiscence of macroscopic charged domains in the bulk that only dissipate over tens of minutes to hours eventually self-healing the device to its initial steady state. Similar recovery behaviour in perovskite thin film (no metal contacts) was also observed by monitoring the recovery of the PL intensity (Fig. 3b) in the dark at a low-power density (where no light-activated meta-stable trap states are formed) after photo-bleaching the photoemission via light soaking (Supplementary Fig. 16). The complete recovery of the PL signal occurs over several minutes to hours. However, the recovery time is dramatically shortened if the film is heated to 47 °C. The quantitative difference in the recovery timescale of the PL for the isolated thin films used for micro-PL spectroscopy to that in devices is attributed to the much higher excitation power density used to create the light-activated trap states, as well as the lack of electrical contacts, which otherwise can expedite the relaxation of these accumulated charges. Furthermore, we observed that the recovery dynamics of the photocurrent is highly temperature sensitive, where a small decrease in temperature to 0 °C almost freezes the recovery, while increasing the temperature from 10 to 70 °C accelerates recovery of the device to <1 min (Supplementary Fig. 9).

Altogether, the observation of light-activated deep-level states along with dielectric constant change and steep temperature dependence is in good agreement with the picture of localized and strongly bound deep electronic trap states activated through light soaking.

### Possible physical origin of photo-degradation/self-healing

Next we systematically explore the following possible mechanisms that could explain the formation of these light-activated trap states: (a) intrinsic defects, (b) ion migration, (c) ferroelectricity and (d) photo-generation of polaronic states enhanced by cation orientational freezing.

First, we consider the possibility of conventional charge trapping by intrinsic defects in perovskite films. The measured timescales for the degradation and recovery processes are, however, much longer (minutes to hours), than typically expected values (nanoseconds to microseconds) observed in inorganic and organic semiconductors. Hence, if the intrinsic defect states were degrading the device, a decrease in the photocurrent should have been observed almost instantaneously after photoexcitation contrary to the slow degradation observed over hours. This is consistent with our observation of a pure bi-molecular recombination process, which is a signature of low-defect density in the large-grain-size perovskites observed during normal solar cell operation and also in the time-resolved PL measurements^24^,^25^. Furthermore recent work by Tian *et al*.^26^,^27^ reported relatively slow timescale (mins) PL enhancement in MAPbI_3_ thin film under continuous light illumination and in different environments. However, for our large-area grain samples, and under our experimental conditions, such behaviour is negligible in the PL time evolution under light soaking (Supplementary Fig. 17). We emphasize that the photo-degradation/self-healing behaviours reported in the present work corresponds to a completely different mechanism than the one reported by Tian *et al*.^26^,^27^. More precisely, these studies report PL enhancement during light soaking and PL degradation when resting perovskites in dark, which is opposite to our observation described in Figs 2 and 3. Moreover, in their study it was found that oxygen was essential for light-curing effect and this phenomenon was significantly more pronounced at crystal surfaces.^26^,^27^ In our photo-stability study of solar cell devices, both the influence of oxygen and surfaces/interfaces are minor effects because of the device and large-grain perovskite structures, which prevent direct exposition to external atmosphere and shrink the number of surfaces/interfaces^24^. Based on the above arguments, the role of intrinsic electronic impurities appears to be a minor mechanism that is not likely to explain the origin of our photo-degradation/self-healing behaviour.

**Figure 4:**
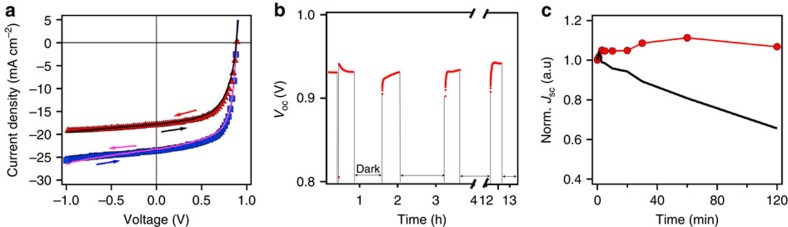
Investigation of possible mechanisms behind the photocurrent degradation. (**a**) *J–V* characteristics before (blue square–pink line) and after 2 h of constant illumination (red triangles–black line); voltages are scanned from forward to reverse (symbols) and from reverse to forward (lines) with delay time of 3 ms (other scan rates are presented in Supplementary Information and do not show hysteresis). (**b**) *V*_OC_ evolution over several light on (red curves) and light off (space in between) cycles. (**c**) Control measurement of time evolution of the photocurrent by applying a forward bias when the device is rested in the dark (red circles) in comparison to the constantly illuminated device (black line).

Previous studies have also attributed the hysteresis and switching effects observed in hybrid perovskites to ion migration that occur at slow timescales^17^,^18^,^20^,^21^,^23^. Therefore, we consider ion migration as one of the possibilities for the observed photocurrent degradation. Different types of measurements such as: (i) changes in the PL and XRD spectra induced by 1-Sun constant light illumination^13^, (ii) *J–V* curve hysteresis under illumination^17^,^18^, (iii) photo-voltage change after light/dark switching cycles^28^ and (iv) field-dependent behaviour^13^,^17^,^18^,^19^,^20^,^23^ have been reported in the literature as benchmarks of ion migration, which could serve as a detection tool for this effect. Thus in order to determine the importance of this effect in our case, we performed these measurements on our perovskite thin film and devices both before and after photo-degradation. The XRD and PL spectra on perovskite thin film after photo-degradation as described earlier do not show significant change. Recent reports^17^,^18^,^19^,^21^,^22^,^23^ have suggested that *J–V* hysteresis in perovskite solar cells is strongly correlated to the migration of ions, which leads to anomalous S-shaped *J–V* curve near reverse bias region^18^,^19^. However, we do not observe any hysteresis in the *J–V* characteristics either before or immediately measured after while light soaking as illustrated in Fig. 4a (see also detailed study in Supplementary Fig. 18). Moreover, the change in the *V*_OC_ is significantly less (40 mV) than those reported in devices (∼200–400 mV) where ion migration was attributed to be occurring^17^,^20^,^23^. Similarly in thin films, we observed no change in the PL emission properties of large grains over several cycles of illumination at device operation conditions and darkness (Supplementary Fig. 19). Moreover, the ion migration-induced hysteresis is independent of light intensity reported by Tress *et al*.^18^ but depends on external electric field^21^ as the mobility of the ions is strongly affected by external fields^17^,^18^,^20^,^21^. To test this, we measure the photocurrent degradation under different light intensity and external electric bias in dark. We find that the percentage of photocurrent decrease is proportional to light intensity (consistent with PL power-dependent curve in Fig. 2) as shown in Supplementary Fig. 9. We also observe that the photocurrent does not change appreciably when an external bias (1 V) is applied in the dark and is illustrated in Fig. 4c (red). These results suggest that ion migration cannot be directly correlated with the observed photocurrent degradation observed in our devices using conventional measurements but is not fully excluded from the possible mechanisms. It is possible that local ionic motion might be the origin for our observed photocurrent degradation/recovery behaviour as suggested by Leguy *et al*.^29^, but experimental evidence in molecular level have to be demonstrated to support this hypothesis. We also note that solar cells in the previous reliability studies^1^,^30^ were made with different precursor stoichiometry and processing conditions, potentially resulting in a slightly different ‘perovskite material' which may explain the observed different behaviour (see also Supplementary Figs 20 and 21 and Supplementary Note 6).

Next we consider the possible role of ferroelectricity that has been proposed to influence charge separation of carriers by formation of local ferroelectric domains^28^,^29^,^31^. It is noteworthy that no direct evidence of a true ferroelectric phase, a paraelectric to ferroelectric phase transition or ferroelectric domains, has ever been unambiguously evidenced in three-dimensional (3D) lead halide perovskites, unlike their 2D counterparts^31^. Moreover, in our study, the observed timescales for photo-degradation and recovery (approximately minutes to hours) do not match the proposed ferroelectric domain migration time (milliseconds)^29^.

Other than the mechanisms discussed above, we propose an alternate physical mechanism for the observed photocurrent degradation and recovery with continuous illumination based on the photo-generation of polaronic states enhanced by cation orientational freezing, which could be relevant for explaining the observed phenomenon (see additional details in Supplementary Note 6). Exploration of a different underlying physical origin of the photo-degradation/self-healing was also motivated by recently published reports suggesting the change in the dielectric constant is associated with structural fluctuations, where photo-induced carriers modify the local unit cell equilibrium and change the polarizability, assisted by the rotational degree of freedom of MA^32^. We also probed this using Raman scattering response before and after creation of the light-activated meta-stable trap states (Supplementary Fig. 22). An increase in the intensity of the Raman modes in the 135–210 cm^−1^ region is observed, which has been reported by Gottesman *et al*.^33^ who also suggest local structural distortion and light-induced local distortions of the structure related to a strong slowing down of the MA motion.

### Theoretical origin of photo-degradation

**Figure 5:**
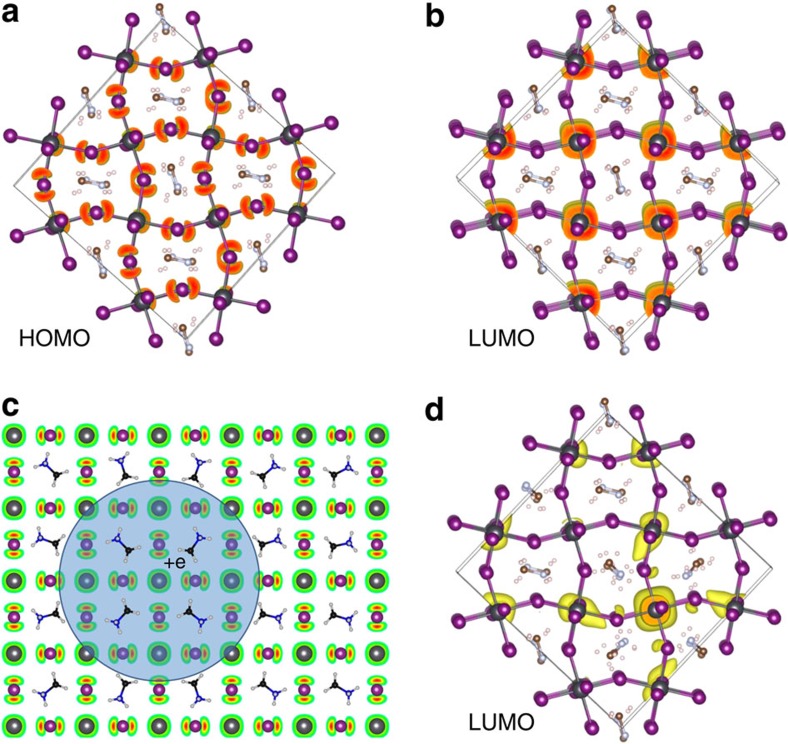
Photo-generation of polaronic states. Crystal structures and charge densities (in yellow) of HOMO (**a**) and LUMO (**b**) for experimental structure calculated using DFT. (**c**) Schematics of a quasiparticle hole (HOMO) dressed by the interaction with neighbouring rotating cations. The blue circle sketches the polaron quasiparticle including its charge (+e). The crystal lattice is composed of the MA (molecules) and the Pb and I (atoms) and we display the charge density of HOMO by red and green colours. (**d**) Localization of LUMO when eight MAs are rotated towards a Pb atom, illustrating formation of a localized electron (polaron). Scheme showing filling of internal traps.

To propose an alternative microscopic origin of the observed photocurrent degradation, we performed symmetry analysis and DFT calculations (Fig. 5). As shown in the symmetry analysis (Supplementary Note 7), Fröhlich-like mechanisms for the long-range coupling of charge carrier to polar vibrational modes are expected to play a role in highly ionic materials as hybrid perovskites. The polar character of the light-induced states is indeed consistent with the enhancement of the dielectric constant under illumination (Fig. 2d). Moreover, acoustic deformation potential is shown to induce additional short-range mechanism for the coupling of charge carriers to the lattice leading to further spatial localization (Supplementary Figs 23–26, Supplementary Table 1 and Supplementary Note 7). The resulting carrier localization can be enhanced by static cation configuration in hybrid perovskites (Fig. 5c). The increase in the near-infrared absorption extending >500 meV deep within the band gap, the local structural distortion probed by Raman scattering, as well as the steep temperature dependence of the photo-degradation are reminiscent of a highly (small) localized polaron picture observed in organic semiconductors^34^,^35^, and previous reports predicting changes in the absorption response over MA reorientation^36^. Similar, charge localization induced by the orientation of the organic molecules^37^,^38^ or coupling between exciton and the lattice^39^,^40^,^41^,^42^ has been proposed by other groups as well. Here we emphasize that the proposed polaron mechanism is not only a matter of charge localization through a fluctuating potential, but also a crossover to a strong coupling regime between the carrier and the lattice. Moreover, as indicated in the present work, polar long-range interactions as well as short-range elastic local field fluctuations play a role in the generation of polarons. These small polarons are expected to have the following attributes: first, the signatures of these localized electronic states are expected to manifest as deep-level trap states within the band gap; second, the formation of these dressed quasiparticles is not expected to be extremely fast, but would still be faster than the timescale observed in the degradation of the large-grain devices. The slow degradation of the device performances is rather attributed to the very slow motion (mobility) and accumulation of these new quasi-static charge states, which coexist with free carriers that lead to the observed photocurrent degradation. Finally, the degradation and recovery of the photocurrent degradation are expected to exhibit steep temperature dependence (activation law). This needs to be elucidated by future studies. For example, further measurements beyond the scope of this study are necessary to definitively assess the small polaron proposition and corroborate its role in the photo-degradation in perovskite devices.

### Practical implications for stable device performance

**Figure 6:**
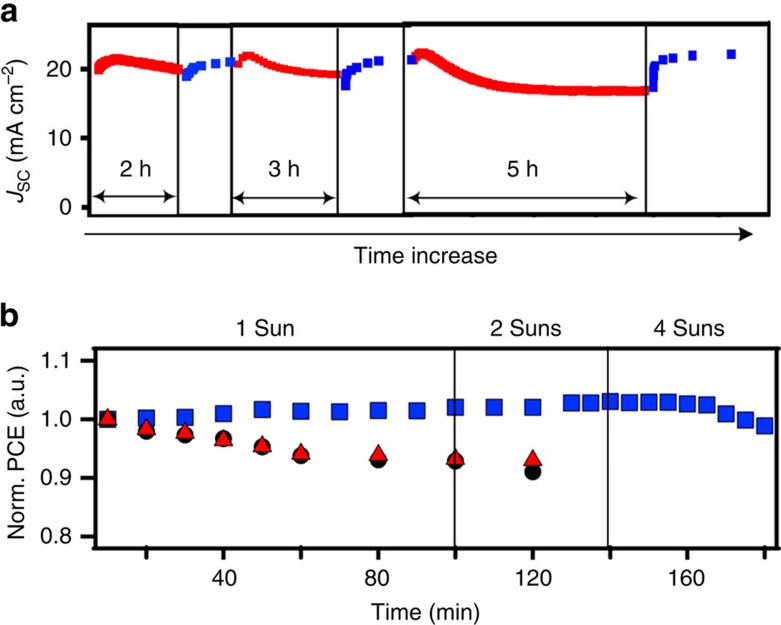
Practical implications for stable and repeatable solar cell performance. (**a**) Multi-cycles of device degradation/recovery over 1 day by turning light on (red symbols) and off (blue symbols). (**b**) Normalized PCE under constant illumination by keeping the device operating at 0 °C (blue squares) and 70 °C (red triangles) as compared with device measured at room temperature (black circles).

Finally, based on our experimental and theoretical analysis we demonstrate that the overall degradation rates can be dramatically controlled by manipulating the solar cell operating conditions. Figure 6a demonstrates the self-healing behaviour of a typical solar cell device measured for few cycles of alternating operations under 1-Sun illumination and in the dark (Fig. 6a). As can be clearly observed, the photocurrent degradation and self-healing is repeatable over multiple cycles of operation and such device restoring behaviour in dark could be one possible approach to mitigate photocurrent degradation. Furthermore, as illustrated in Fig. 6b, we demonstrate that the device efficiency under constant illumination can be significantly impeded by lowering the temperature down to 0 °C, up to 4-Sun illumination intensity as no photocurrent degradation is observed in contrast to devices at higher temperature.

In summary, we attribute the observed reduction in the overall efficiency with constant illumination in crystalline large-grain-size hybrid perovskite solar cells to the formation of light-activated meta-stable trap states. Structural, electronic and optical spectroscopies are examined to discuss the role of intrinsic traps, ion migration and ferroelectric domain formation as the physical origins of these light-activated states. Theoretical predictions and preliminary spectroscopic characterizations lead us to propose an alternative physical origin of these states to the formation of spatially localized charge states (or small polarons) deep within the band gap. These charged states coexist with the conventional photo-generated free carriers, dominating the photocurrent in all regimes, ultimately resulting in photo-degradation of the devices. Based on the scientific understanding gained from our investigation, we suggest that the formation of these light-induced defects can be controlled to obtain photo-stable perovskite photovoltaic devices by preventing the meta-stable electronic state formation and accumulation (temperature), or by alternating the light on/off cycles for practical application. These investigations are the first step towards establishing the principles towards reliable and stable perovskite-based photovoltaic devices.

## Methods

### Perovskite solar cells

The planar solar cells are in the structure of ITO/PEDOT:PSS (Clevis 4083)/large-grain Perovskite/PCBM/Al (Fig. 1a), the active layer is made using our previously developed hot-casting method^24^ with PbI_2_ and MACl (1:1) precursor in DMF and top contact layer is fabricated by spin casting 20 mg ml^−1^ PCBM in chlorobenzene followed by thermal evaporating aluminium in vacuum chamber. The completed solar cell is mounted into a cryostat and pumped down to 10^−4^ torr for device measurement under constant AM 1.5 white light solar simulator. The perovskite thin films for optical measurement are prepared on glass substrate using the same method as described above.

### PL and optical absorption spectroscopy

Spectrally- and time-resolved PL spectroscopy were performed with a home-build microscopy set-up focusing a 640-nm radiation pulsed laser beam (∼6-ps pulse duration) close to the diffraction limit with a × 50 (numerical aperture=0.45) long-working distance objective (resolution ∼0.6 μm at 640 nm). In this study, all spectroscopy experiments were conducted by probing a single large-area crystalline grain (>50 μm size) of the organometallic perovskite thin films (see also the study by Nie *et al*.^24^ for comparison of large and small grains properties). PL spectra were obtained through a spectrograph (Spectra-Pro 2300i) and a CCD camera (EMCCD 1024B) yielding a maximum error of 0.2 nm on the emission spectra. Time-resolved PL measurements were performed by means of a time-correlated single-photon-counting technique with a silicon avalanche photodiode. Absorption spectroscopy was performed using a super-continuum fibre laser and measuring both reflected and transmitted beam spectra (see details in Supplementary Information section 4). The linear extinction spectra in the near-infrared region were measured using a high-brightness, Fourier transform-based spectroscopic technique, described previously^43^. If not stated otherwise, all spectroscopy measurements took place at room temperature and the thin films were kept in vacuum <10^−5^ torr.

### Calculations

All theoretical computations presented are performed using the Vienna *ab initio* simulation package (VASP)^44^,^45^ based on DFT with the all-electron projected augmented wave (PAW)^45^ method using the PBE exchange correlation functional^42^. The distributed PAW potentials have been generated by Kresse following the recipes discussed in ref. 45. Electron-ion interactions are described using a kinetic energy cutoff of 525 eV. Using a larger cutoff of 900 eV results in negligible changes in the band gaps (<1 meV) and the same orbital localization pattern is found. Moreover, inclusion of van der Waals corrections to the DFT functional proposed by Dion *et al*.^46^ and implemented into the VASP software^47^,^48^ lead to the negligible changes in the band gaps and orbital spatial extend in several test cases. Valence states included the Pb 5d, 6s and 6p states, I 5s and 5p states, C 2s and 2p states, N 2s and 2p states, and the H1s state. We perform Brillouin-zone integrations using Monkhorst–Pack grids of special points with (4 × 4 × 4) meshes for the calculation of the structural and electronic properties. All calculations were done with and without spin-orbit coupling, and the overall conclusions of the results remained unaffected.

### Data availability

All the data that support the findings of this study are directly available from the authors on request.

## Additional information

**How to cite this article**: Nie, W. *et al*. Light-activated photocurrent degradation and self-healing in perovskite solar cells. *Nat. Commun.* 7:11574 doi: 10.1038/ncomms11574 (2016).

## Supplementary Material

Supplementary Information Supplementary Figures 1-26, Supplementary Table 1, Supplementary Notes 1-7 and Supplementary References
