# Supplementary material for: Light-activated photocurrent degradation and self-healing in perovskite solar cells
Source: Nat Commun. 2016 May 16;7:11574. doi: 10.1038/ncomms11574 (PMC4873646; doi:10.1038/ncomms11574)
Supplement: Supplementary Information — Supplementary Figures 1-26, Supplementary Table 1, Supplementary Notes 1-7 and Supplementary References [file ncomms11574-s1.pdf]

Supplementary Information

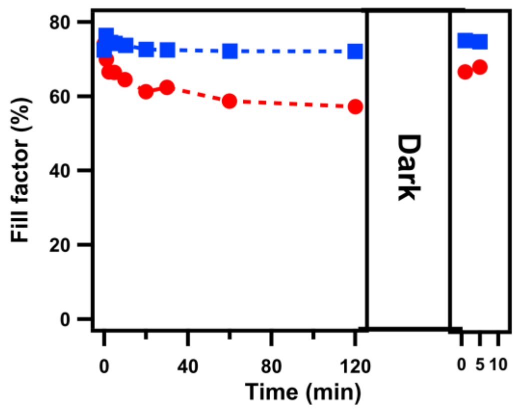

Supplementary Figure 1. Time evolution of the fill factor of the device presented in Fig. 1 over 2-hours under constant 1-sun illumination.

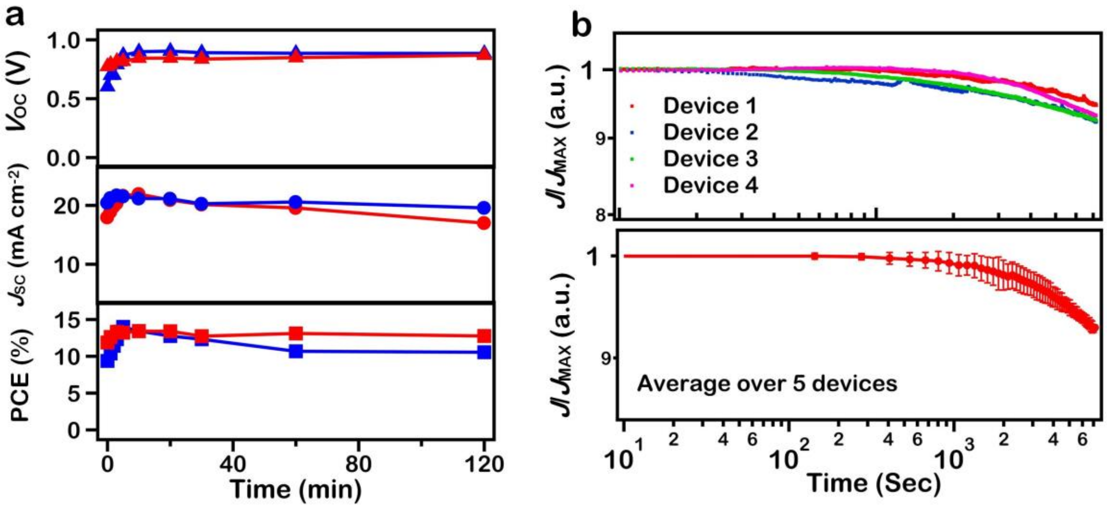

Supplementary Figure 2. Statistics of the photo degradation process over several devices. (a) Study of the photo-degradation of the figures of merit for two additional solar cell devices. Device 1 and 2 are sketched in blue and red, respectively. **(b)** Photocurrent degradation for 4 additional devices (upper panel) and the average photocurrent degradation over 5 devices with error bars.

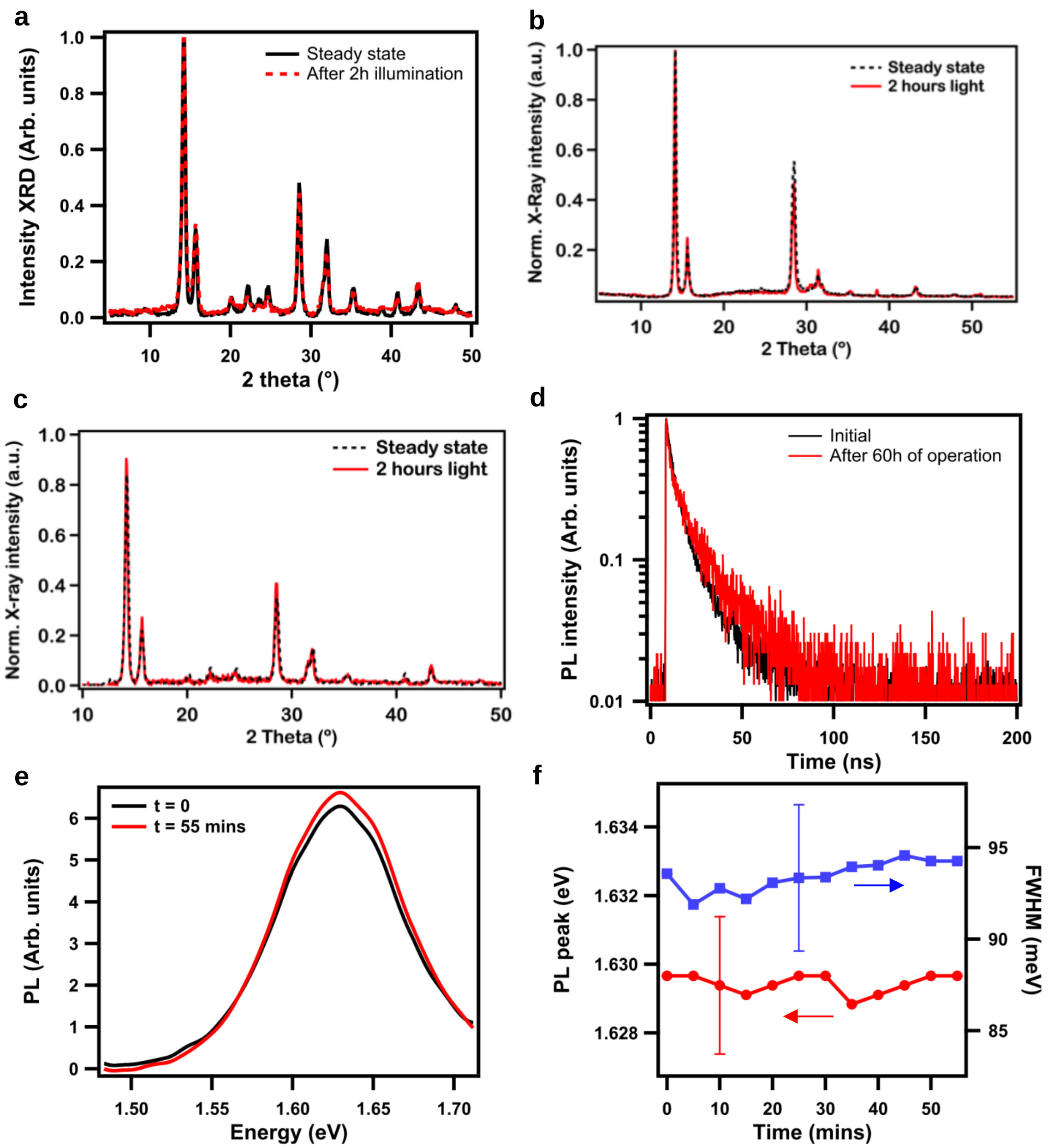

Supplementary Figure 3. Structural and chemical stability under constant illumination of perovskite thin films. (a-c) X-Ray diffraction pattern of three different samples acquired before and after 2 hours under 1-sun constant illumination in vacuum. **(d)** Time-correlated single photon counting histogram of the PL after synthesis (black) and after more than two days under illumination at device operation conditions (red). **(e,f)** Time evolution of the photoluminescence properties of perovskite thin films under constant illumination for 60 mins. Error bars correspond to the minimum resolution of our photoluminescence spectroscopy system.

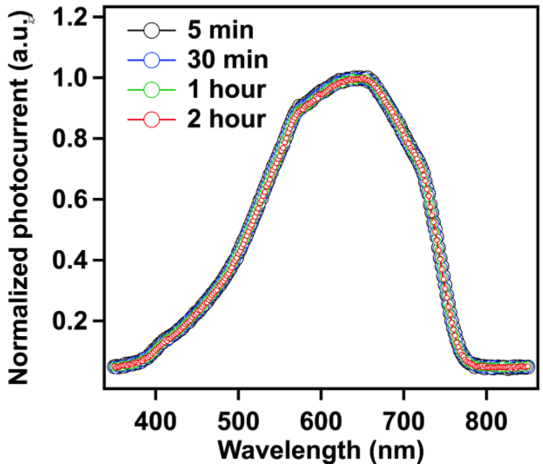

Supplementary Figure 4 . Time evolution of the photocurrent spectrum in a solar cell device under constant 1-sun illumination.

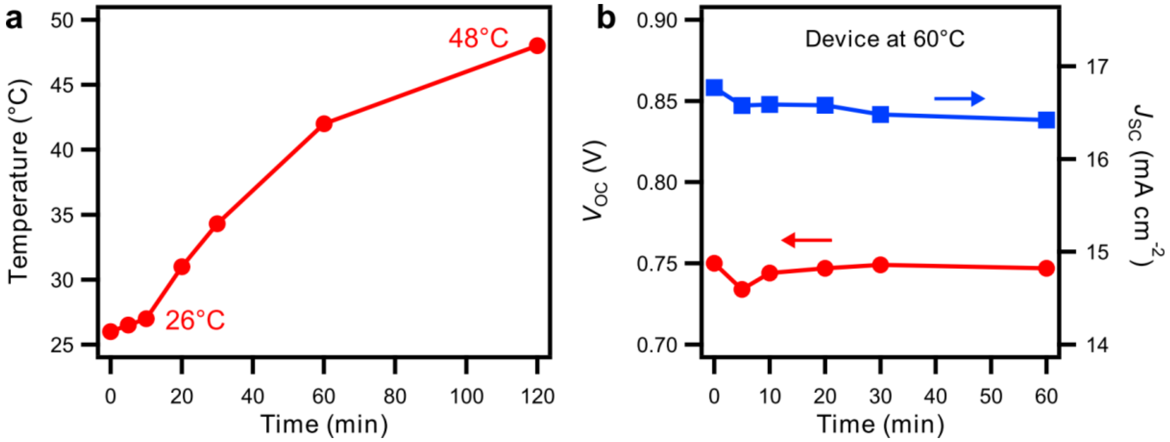

Supplementary Figure 5. Effect of sun-light-activated heating on solar cell performances. (a) Temperature change at the device surface as a function of time under constant 1-sun illumination. **(b)** V_{OC} and J_{SC} of the encapsulated solar cell kept at 60ºC in dark and measured under 1-sun illumination every 10 min.

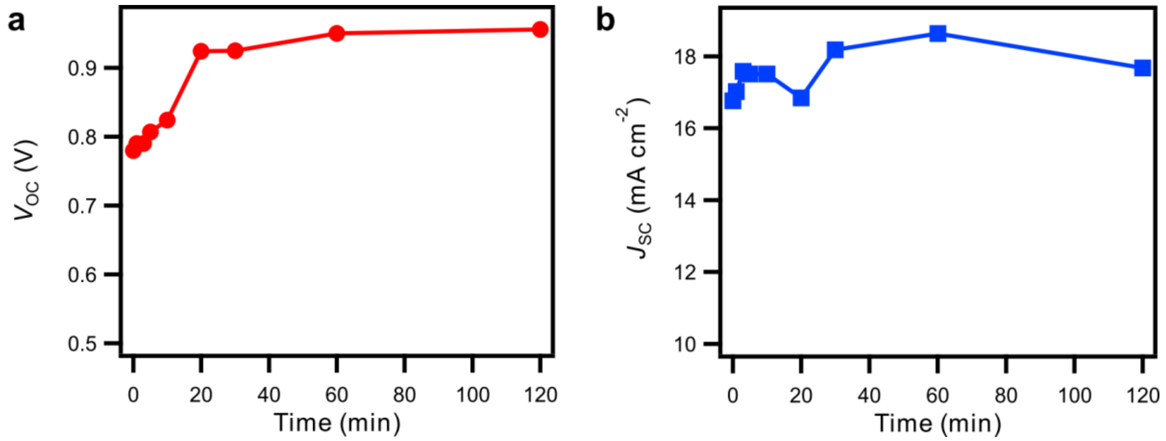

Supplementary Figure 6. Time evolution of a solar cell figures of merit under forward bias in dark. (a) Open circuit voltage and **(b)** short circuit current density. The device is constantly biased at open-voltage (~0.9 V) in dark (flat band condition) and *J-V* characteristics are sporadically measured under 1-sun light.

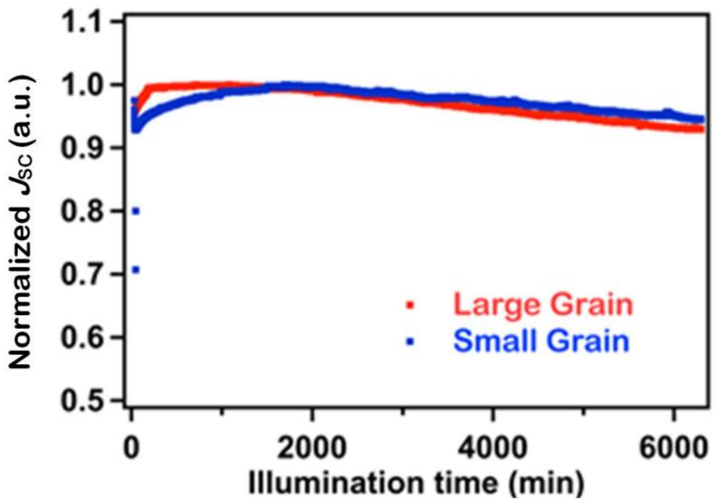

Supplementary Figure 7. Time-evolution of the photocurrent for large-grain-based (red) and small-grain-based (blue) solar cell devices stressed at short circuit conditions and under 1-sun constant illumination.

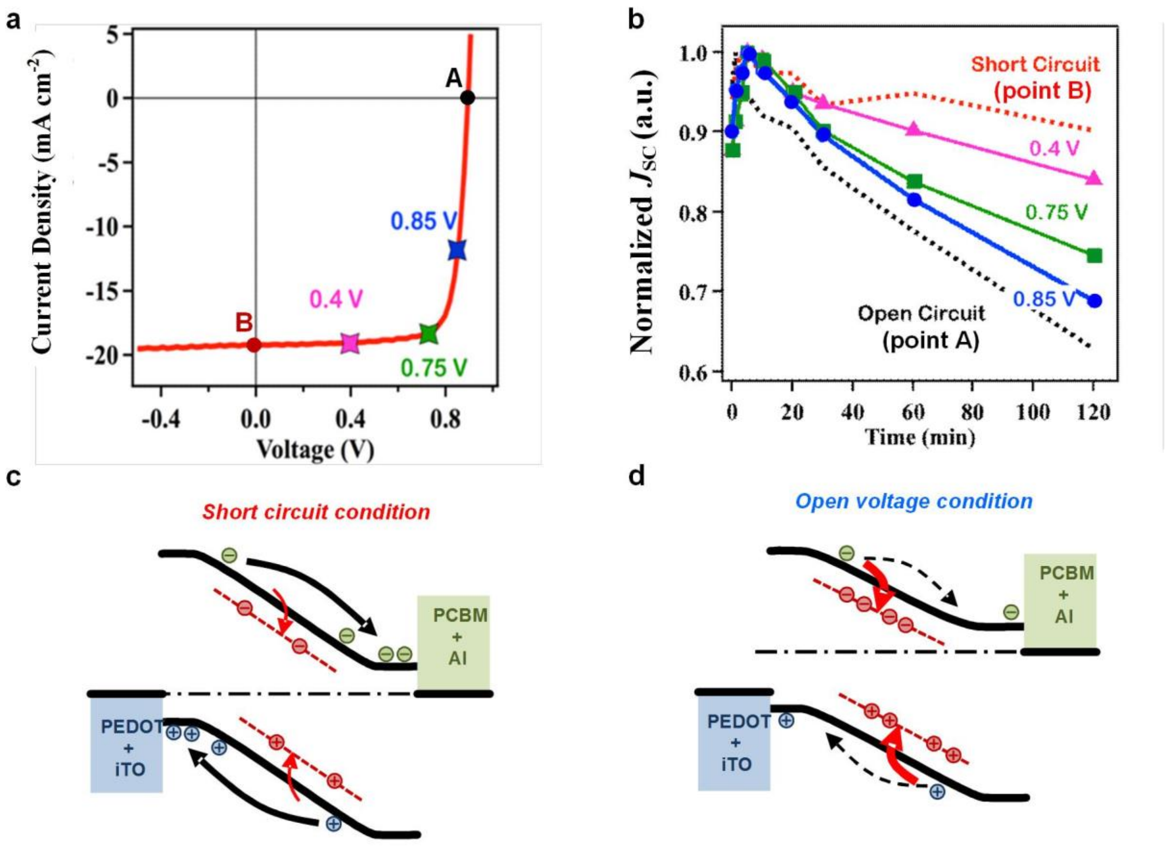

Supplementary Figure 8. Photocurrent degradation under 1-sun illumination for solar cells stressed at different load points. (a) *J-V* characteristics and sketch of the load points. (b) Time-evolution of the photocurrent for devices under constant 1-sun illumination. (c,d) Schematics of band structure in our solar cell devices at short circuit and open voltage conditions. According to Fig. 1f in the MS, the red dotted lines refer to light induced meta-stable trap states that relax in dark returning the device to its equilibrium state. Arrows indicate how photo-generated carriers can transfer and populate those states under light with different probability depending on the device load point.

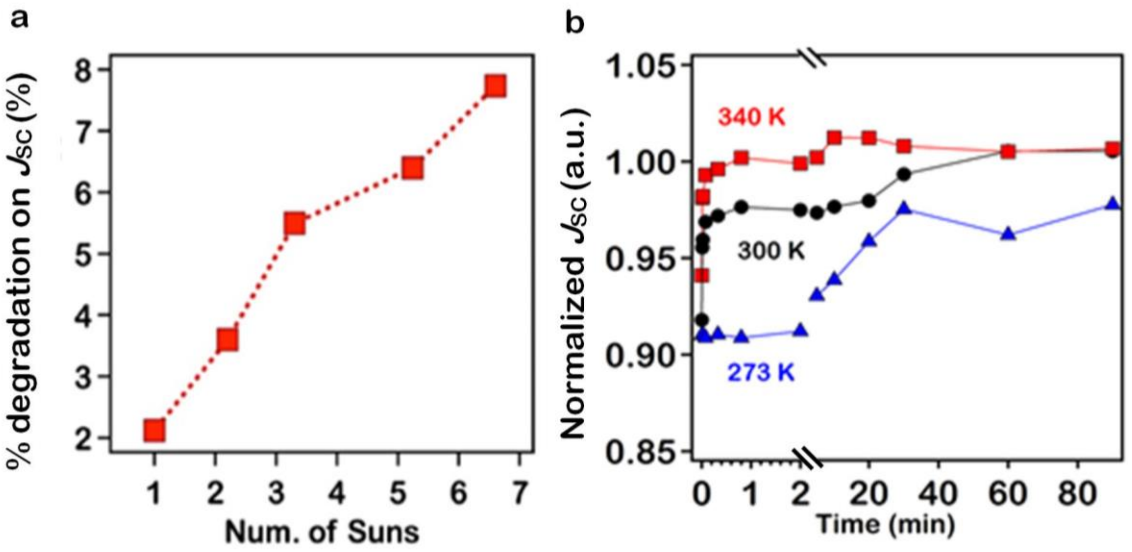

Supplementary Figure 9. (a) Light excitation power dependence of the photocurrent degradation. Percentage of J_{SC} degradation after 1 hour when monitoring device photocurrent at short circuit condition and constantly illuminating it with various sun powers. **(b)** Temperature dependence of the photocurrent recovery process.

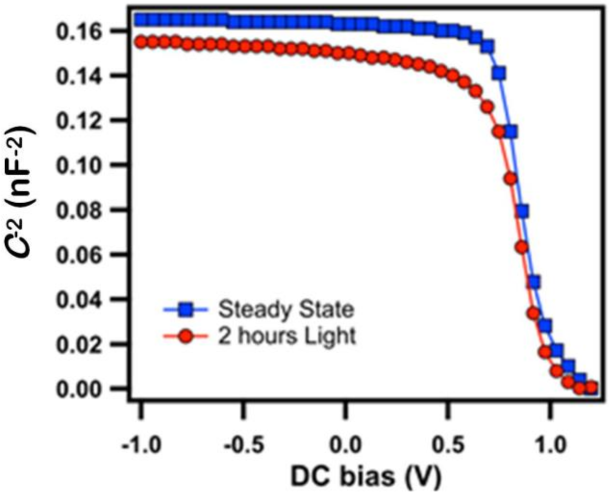

Supplementary Figure 10. *C-V* characteristics time-evolution after constant 1-sun illumination for 2-hours for the solar cell stressed at open circuit condition. The measurement was taken using 0.02 V AC RMS voltage at a frequency of 100 kHz.

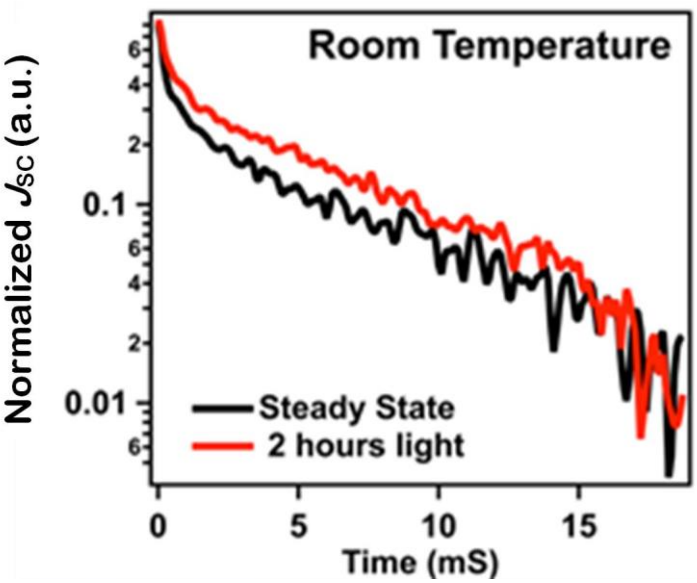

Supplementary Figure 11. Photocurrent transient dynamic for a solar cell device stressed at V_{OC}. At time zero the voltage is quickly switched from open voltage to short circuit conditions and the transient photocurrent is then monitored. The black curve refers to device at steady state and red curve shows same device after 2 hour illumination.

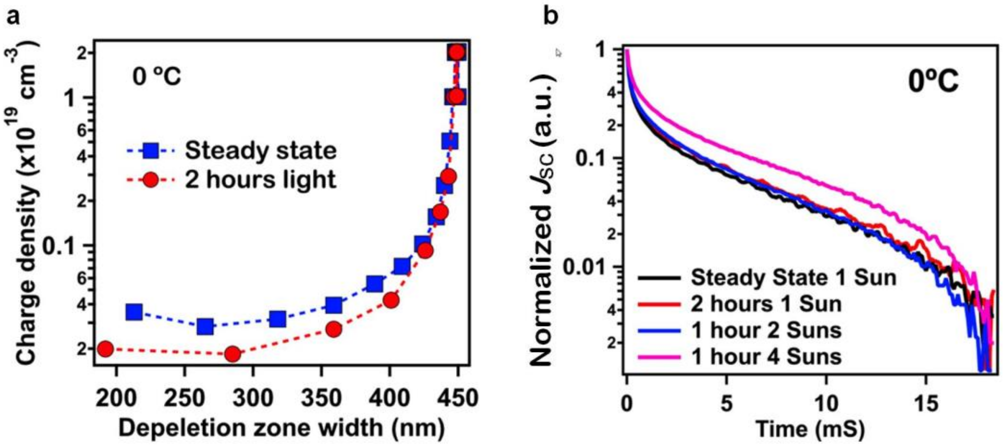

Supplementary Figure 12 . Proof of blocking of light-activated trap states at 0 °C. (a) Charge density profile extracted from *C*-*V* measurement before and after 2 hour illumination at open circuit condition. (b) Photocurrent transient when device is switched from open circuit to short circuit condition at room temperature and at 0 °C under constant illumination.

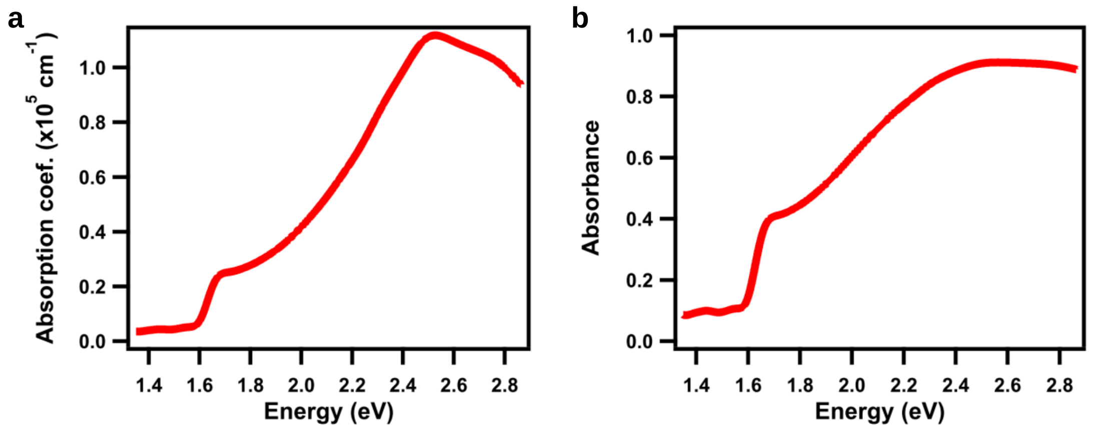

Supplementary Figure 13. Absorption properties. Absolute absorption coefficient **(a)** and absorbance **(b)** of a single large-area grain perovskite in a 280-nm-thick thin film.

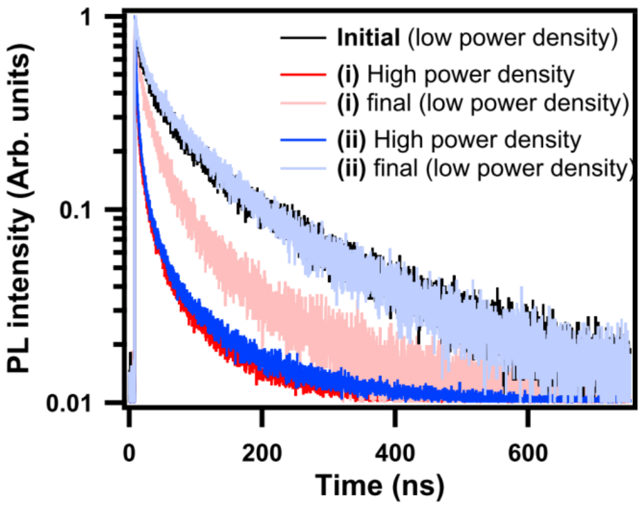

Supplementary Figure 14 . Time-correlated single photon counting histogram of the PL for different light excitation power density for (i) continuous light soaking and (ii) with recovery in dark (by resting the sample in the dark between each data point). The dark curve serves as reference and corresponds to the signal response of the perovskite thin film freshly prepared and is measured under very low excitation photon power density (0.9 $\mu J/cm^2$) to prevent the formation of the light-activated meta-stable trap states. After a power density increase/decrease cycle, the thin film response is measured again at low excitation power density. The 'high photon fluence' corresponds to an excitation power density of 125 $\mu J/cm^2$.

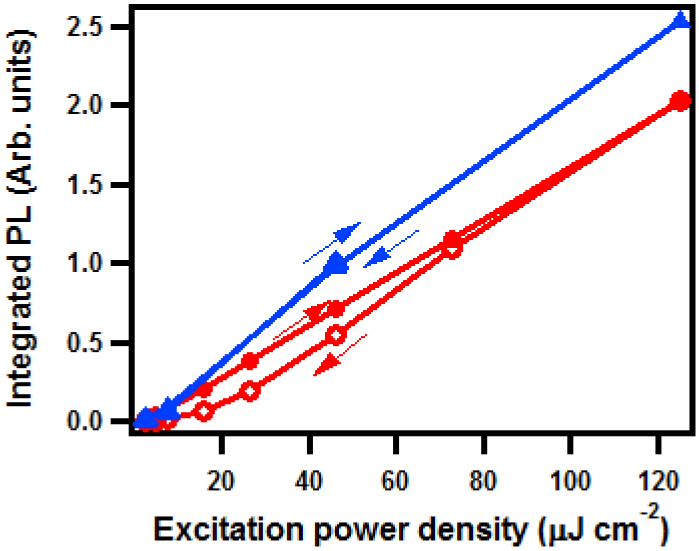

Supplementary Figure 15. Excitation power density dependent integrated photoluminescence for (red) continuous light soaking and (blue) with delay in dark (by resting the sample in the dark between each data point). Filled and open symbols stand for excitation power density increase and decrease cycle, respectively. Lines between symbols are guides for the eye.

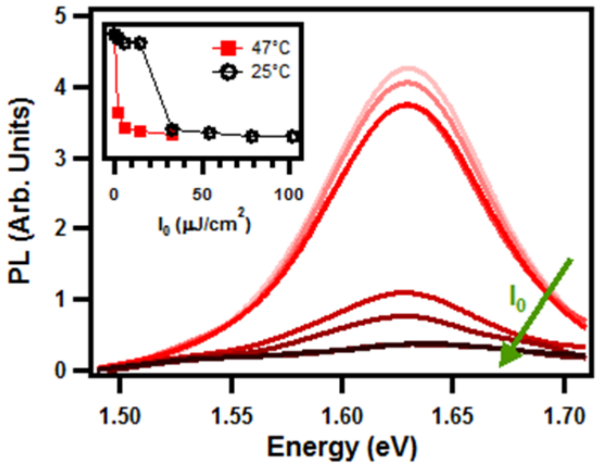

Supplementary Figure 16 . Photoluminescence photo-bleaching at room temperature after light soaking with various power density I_0 at room temperature (25°C). (inset) PL amplitude after light soaking at different power densities showing the photo-bleaching power density threshold.

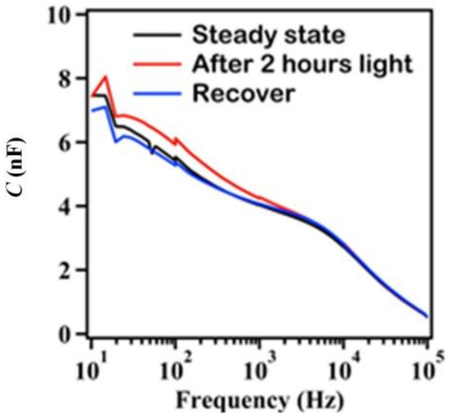

Supplementary Figure 17. Capacitance spectrum as a function of AC field frequency for solar cell device at the steady state (black), after 2 hours illumination (red) and after recovery (blue).

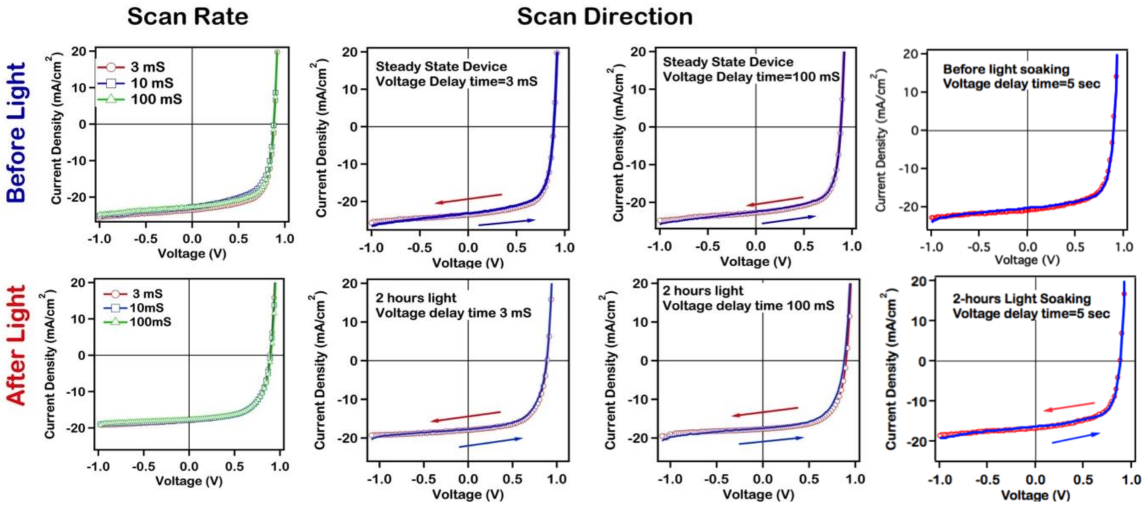

Supplementary Figure 18. Hysteresis test by scanning *J-V* curves under one sun for devices before (upper panel) and after constant illumination for 2 hours (lower panel) under open circuit condition with different voltage scan rate.

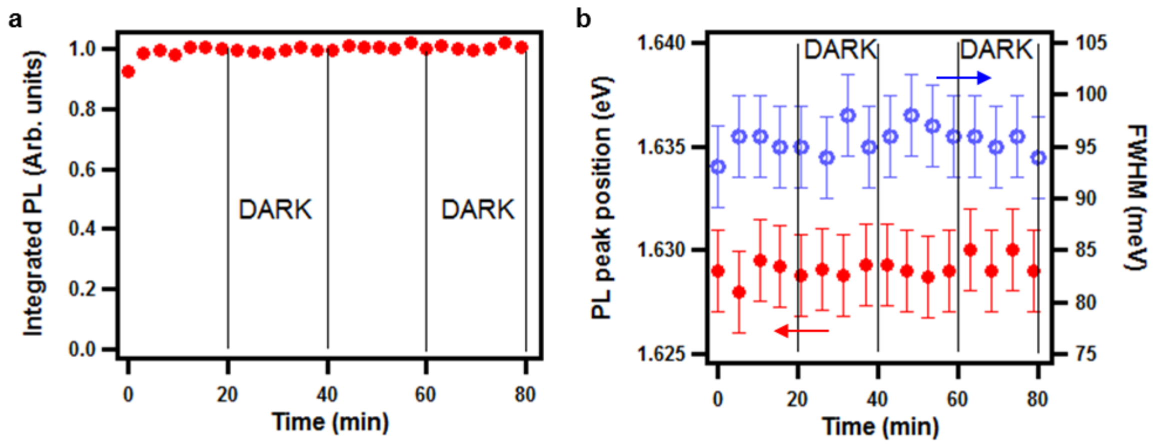

Supplementary Figure 19. Photoluminescence characteristics of a perovskite thin film over several cycles of light soaking (constant illumination, yellowish regions) and rest in dark (grey region). **(a)** Integrated photoluminescence. **(b)** PL peak position and full-width-at-half-maximum (FWHM). Error bars correspond to the minimum resolution of our photoluminescence spectroscopy system.

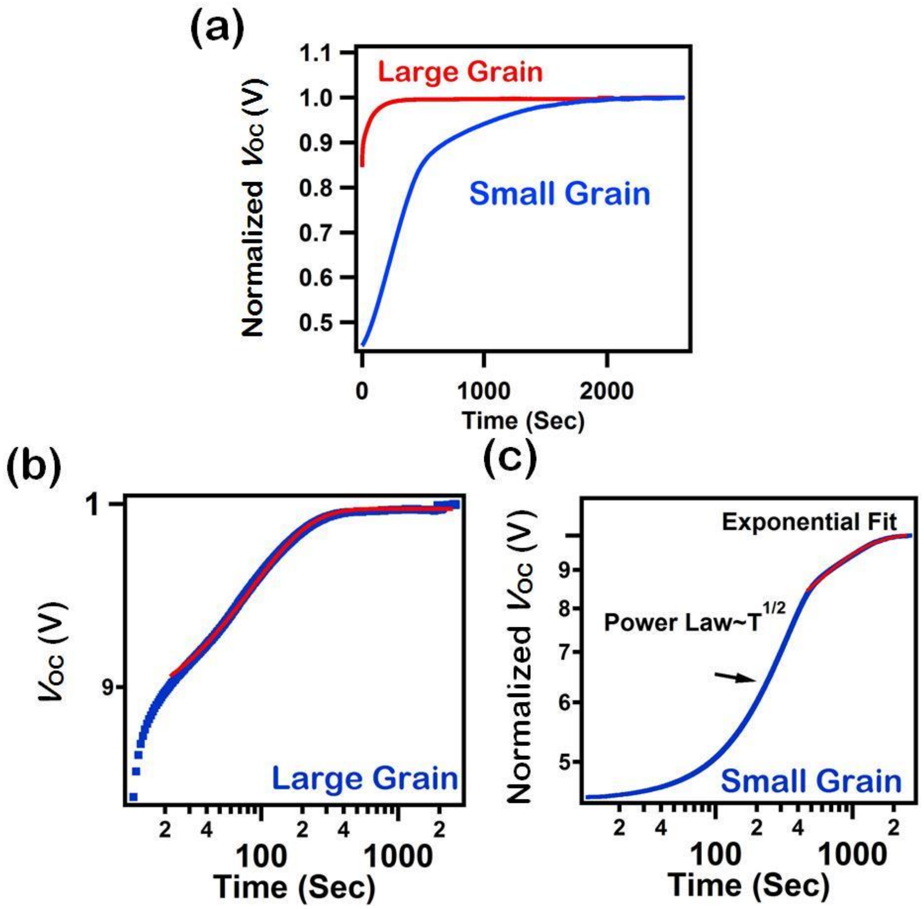

Supplementary Figure 20. Impact of ion migration on open circuit voltage. Open circuit voltage for **(a)** initial increase upon shining light for large grain device (red) and small grain device (blue), **(b)** large grain device with exponential fit, **(c)** small grain device with power law fit in short time and exponential fit over long time scale.

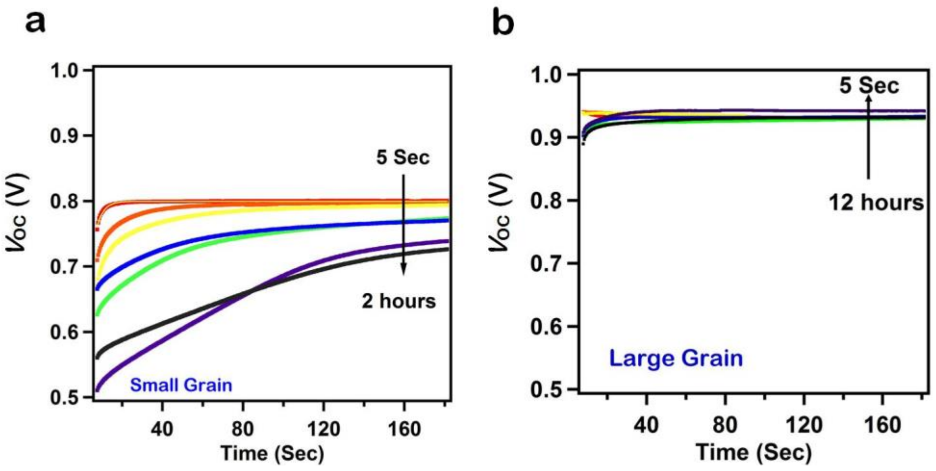

Supplementary Figure 21. Open circuit voltage time evolution for **(a)** small grain device

and **(b)** large grain in light after resting the device for various time periods

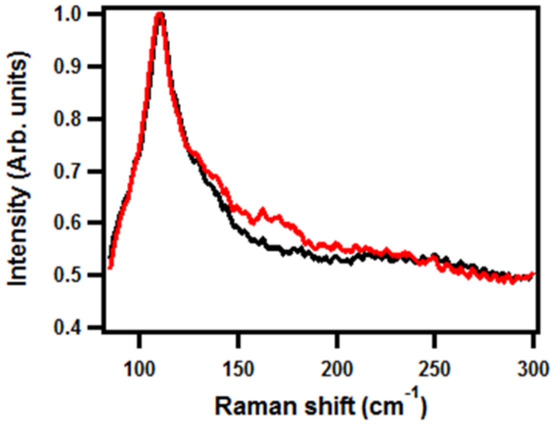

Supplementary Figure 22. Raman spectra acquired for the pristine perovskite film (black) and after photo-degradation (red).

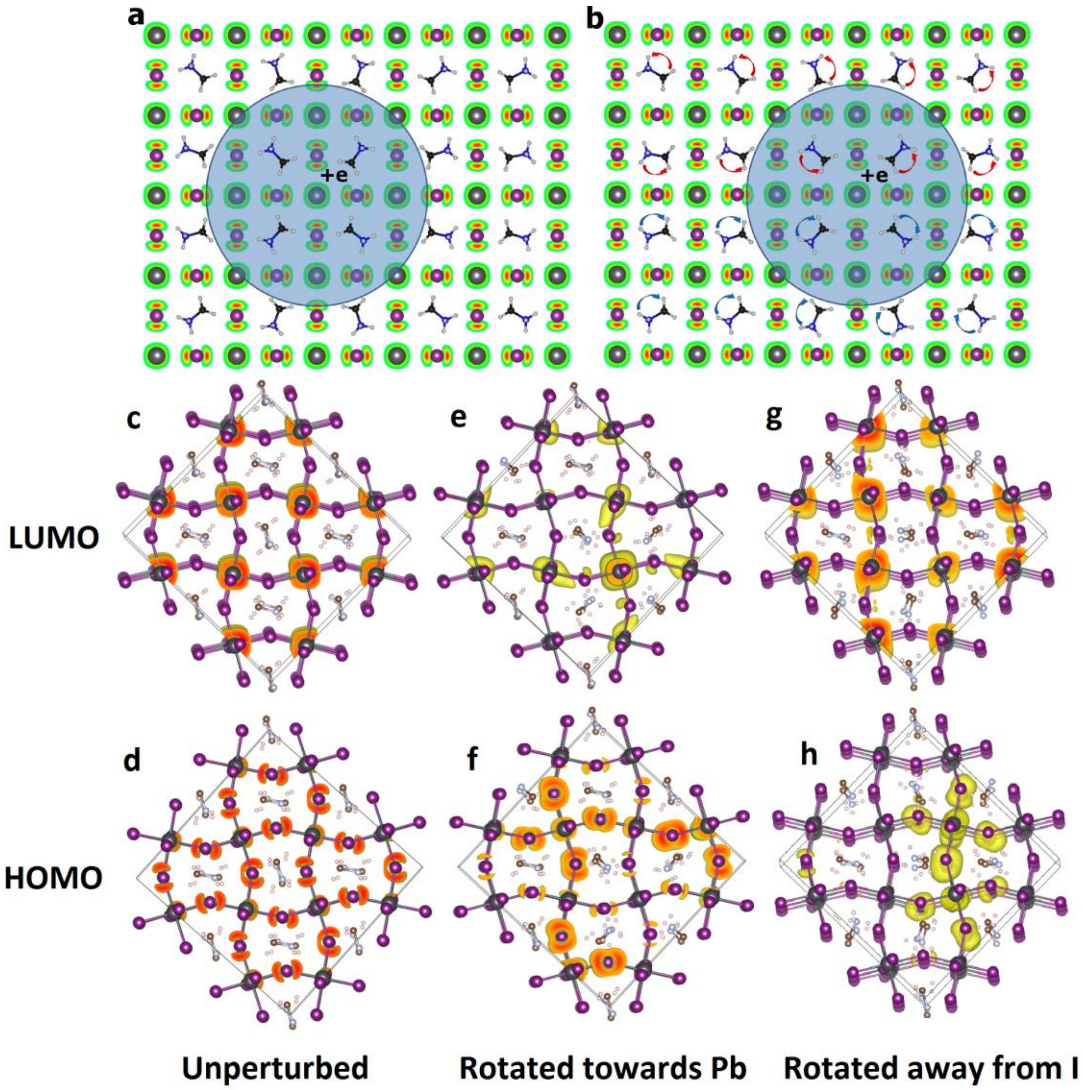

Supplementary Figure 23. Localization due to rotation of MA. (a,b) Artist view of a quasiparticle hole dressed by the interaction with neighboring rotating cations and travelling across the crystal. Crystal structures and charge densities including SOC of HOMO (VB maximum) and LUMO (CB minimum) for **(c, d)** experimental structure, **(e, f)** when 8 MAs are rotated towards a specific Pb atom, and **(g,h)** when 12 MA's are rotated away from a given I atom, illustrating formation of electron and hole polarons related to the dipole arrangement of the methyl ammonium.

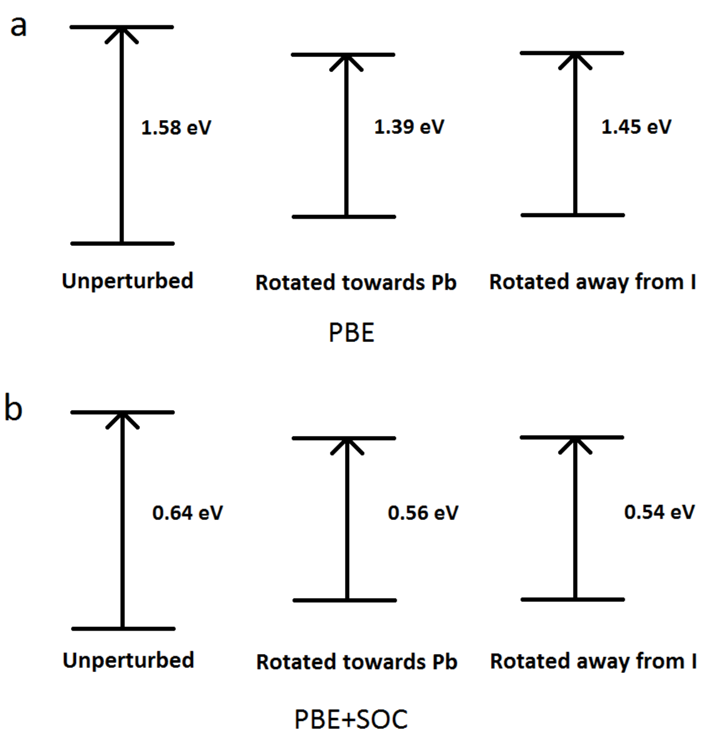

Supplementary Figure 24 . Illustration of band gaps for the unperturbed system, the system rotated towards Pb, and the system rotated away from I using **(a)** PBE and **(b)** PBE+SOC.

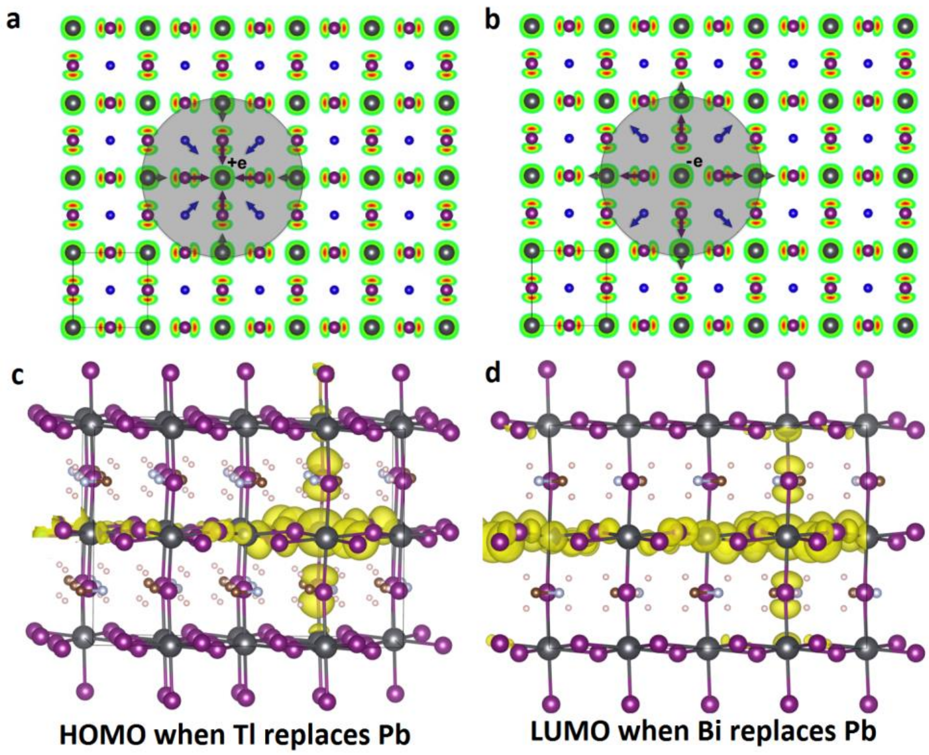

Supplementary Figure 25. Localization due to volumetric strain. Artist's view of the volumetric strain surrounding a localized **(a)** hole or **(b)** electron. **(c)** localized HOMO in the optimized structure where a Pb atom (+2 cation) has been replaced with a Tl atom (+1 cation). **(d)** localized LUMO in the optimized structure where a Pb atom has been replaced with a Bi atom (+3 cation)

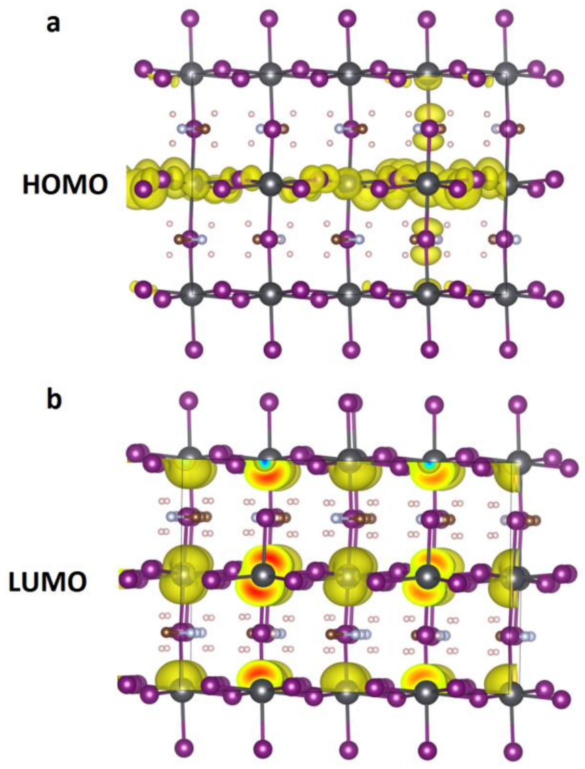

Supplementary Figure 26. Localized HOMO in a structure that contains all Pb atoms, but is using the coordinates obtained from a present Bi atom in place of one of the Pb atoms.

	Equitorial I	Apical I	C	N
Bi	-10	3.5	-1	1
Tl	3	1.5	0	-1

Supplementary Table 1. Displacement of atoms immediately surrounding the centrally replaced Pb after optimization. Displacements are given in pm.

Supplementary Note 1. Statistics of photo-degradation over several devices

To further validate our measurements, we have performed for 2 additional devices the same measurements of photo-degradation as in the main text (Supplementary Figure 2). Identical to what is reported in the main text, the V_{OC} do not change over time once it reaches the steady state value, the only degradation parameter is the J_{SC} and thus leads to PCE change. Moreover, we investigated the photocurrent photo-degradation in four additional devices (Supplementary Figure 2). The average J_{SC} photo-degradation over all measured devices is 8% of its original value (Supplementary Figure 1b, lower panel).

Supplementary Note 2. Evidence of chemical and structural phase photo-stability

X-ray diffraction

First, we excluded light-activated structural phase transition and chemical change in the perovskite material by comparing the X-ray diffraction patterns of the same thin film before and after 2-hours under constant 1-sun illumination in vacuum (Supplementary Figure 3a-c). In both cases we observe identical perovskite main diffraction peaks at 14.2º (110) and 28.5º (220) indicating the crystal structure is not affected by 1-sun light illumination over several hours. An additional weak diffuse scattering contribution appears below the main Bragg peaks that are consistent with the build-up of heterogeneous local lattice strain. We notice that there is diffusive scattering in the presented XRD spectra around 12° in Supplementary Figure 3a, we think that this might not be representative of the several other spectra that we have measured over the several additional samples as shown in Supplementary Figure 3b,c, we do not see any appreciable difference in the PbI_2 signature.

Based on these, we suspect that the observed change in XRD at 12° is possibly due to the testing condition of XRD, which was performed, in this particular case, in ambient air.

Photoluminescence spectroscopy

Second, structural and chemical photo-stability were also verified by time-resolved (Supplementary Figure 3d) and spectrally-resolved (Supplementary Figure 3e,f) photoluminescence measurements of perovskite thin films. In the former case, the PL decay response measured under low excitation power density shows no significant change after 60-hours under identical-to-solar-cell illumination operation conditions. Besides, the PL spectrum also presents no significant change in the PL peak properties – position, full-width-at-half-maximum, and amplitude – as a function of time of illumination at low power density.

Photocurrent spectrum

Finally, the device photocurrent spectrum was tested in AC mode (from monochromatic light chopping at 100 Hz) at short circuit while it is biased with constant DC AM1.5 1-sun illumination as a function of time (Supplementary Figure 4). Over time we observed photocurrent reduction in magnitude that is consistent with J_{SC} measurement with light *J-V* characteristics. However, the photocurrent spectra remain identical when normalized as plotted in Supplementary Figure 4. Therefore, the degradation observed by J_{SC} over time does not alter the spectral shape of the photocurrent, and this is additional evidence that long time illumination does not degrade the crystal structure.

In summary, in this section we have demonstrated that we do not detect any light-activated structural phase transition, as verified by X-Ray diffraction (XRD), photoluminescence spectroscopy, and spectrally-resolved photocurrent

Supplementary Note 3. Exclusion of other possibility at the origin of photocurrent degradation

In order to validate our hypothesis on the origin of photo-stability in large-grain-based organometallic perovskite solar cells, we did control experiment in dark to excluded a) heating effect and b) bias effect over device stability.

Excluding the heating effect in dark

We notice under normal operating conditions, there is a temperature increase of about 22 ºC by 2-hour constant 1-Sun illumination at the surface of the solar cells (Supplementary Figure 5a), measured every 10 minutes via a K-type thermal coupler positioned right on top of the testing device. In order to examine impact of heating towards device stability within that range, we heat the device in dark at 60ºC and measure the solar cell performances every 10 min in light (<1 min measurement) to monitor the change of both V_{OC} and J_{SC} (Supplementary Figure 5b). As a result, both V_{OC} and J_{SC} remain unchanged, thus the results clearly confirm that the temperature is not responsible for the changes in device performances over time under constant 1-sun illumination as reported in the main text.

Excluding the effect of forward bias in dark

We further verify that the photo-degradation observed under constant illumination is not impacted by voltage, *i.e.* by the bias voltage applied to the devices, or the internal photo-voltage under open circuit condition. In other words, the solar cell figures of merit were sparsely monitored under 1-sun light while otherwise keeping the device stressed at flat band condition (forward 0.9 V bias) in dark. Both V_{OC} and J_{SC} show no degradation for two hours after reaching their steady state in the first ~20 minutes (Supplementary Figure 6).

These results demonstrate that *J-V* scans do not have an impact on the device performances and the observations reported in the main text are primarily light-activated effects.

Photocurrent degradation for different perovskite recipes

We also verified that the photocurrent J_{SC} degradation is independent of crystal structures in organometallic perovskite-based solar cell devices. Supplementary Figure 7 compares the photocurrent degradation in our devices (i.e. showing large-area grains, >100 μm) and a device presenting small grains (~0.1-0.5 μm) obtained using $PbCl_2$ and MAI (1:3 molar ratio). Both types of devices exhibit similar decrease trends in J_{SC} (about 8% drop) after 2-hours under constant 1-sun illumination. Similar results are also reported in pure halide perovskite systems by Unger *et al.*[19].

Supplementary Note 4. Photocurrent degradation/recovery in solar cells

In this section, we present results complementary to those reported in Figures 1 and 2 of the main manuscript that we feel important for the readers and the understanding of our work.

Fill factor degradation under constant illumination

As complementary of the figures of merit reported in Fig. 1c-e, we derived the corresponding fill factor during the first cycle of space charge accumulation (Supplementary Figure 1).

Photocurrent degradation for devices stressed at different load points

Although this report focuses on the degradation of performances in solar cells devices stressed at either open-voltage or short-current conditions (see main text), we performed similar studies for other points along the J-V characteristics (Supplementary Figure 8a). As

expected, over 2-hours under constant 1-sun illumination the photocurrent J_{SC} degrades more or less rapidly depending if the device stress point is closer to, respectively, the open-circuit operation point or the short circuit one (Supplementary Figure 8b). This observation corroborates the possibility of extracting charges trapped (or inversely to accumulate more space charges) on the light-activated meta-stable trap states (or polaron states) more-or-less easily depending on the band-alignment schemes at the different operation points in solar cell devices (see sketch in Supplementary Figure 8c,d).

Power dependence of photocurrent degradation and temperature dependence of photocurrent recovery

We also measured the percentage of degradation in the J_{SC} of the solar cell as a function of light excitation power (or number of suns) (Supplementary Figure 9). The solar cell performances degrade almost four times faster under a constant illumination intensity of seven sun as compared to the standard one sun photo-excitation (J_{SC} degrades by 2.12% at 1 sun up to 7.6% at 6.6 suns) indicating that the density of light-activated trap states is directly proportional to the light excitation power density as observed in the photoluminescence data.

Light-activated space charge accumulation in solar cells verified by C-V measurements

The charge density profiles reported in Fig. 2a of the main text are obtained from the C-V characteristics in Supplementary Figure 10. From the C-V measurements the charge density profile $N_d(x)$ is derived by[49–51]

$$N_d(x) = \frac{2}{\varepsilon_r \varepsilon_0 A^2}\left[\frac{d}{dV}\left(\frac{1}{C^2}\right)\right]^{-1} \qquad \text{(Supplementary equation 1)}$$

where x is the depletion width determined by

$$x = \varepsilon_r \varepsilon_0 A / C$$ (Supplementary equation 2)

ε_r is relative dielectric constant calculated from the capacitance of the device at zero bias (~20), and A is the device active area (0.035 cm^2), and ε_0 is the vacuum permittivity.

Transient photocurrent at room temperature

The photocurrent transient measurements also support the hypothesis of the formation and accumulation of charges at the light-activated meta-stable trap states. This method probes the device discharging time (equivalent to RC time constant) when it is abruptly switched from an open-circuit condition to short circuit condition. The discharging time significantly increases after the solar cell is constantly illuminated for two hours as compared to its steady state (Supplementary Figure 11) suggesting the presence of light induced charges within the device either in the perovskite film or at the perovskite/doped-layer contacts.

Charge density and photocurrent transient in solar cells at 0 °C

To support our observation in Fig. 5b that the creation of light-activated trap states or polarons are inhibited when operating the solar cell at 0 °C, we again investigated the devices charge density profiles and transient photocurrent. The space charge density slightly decreased after two-hours illumination at 0 °C as compared to the initial state (Supplementary Figure 12a). Similarly, at 0 °C the photocurrent transient measurements demonstrated that the discharging lifetime increases after one hour constant illumination (Supplementary Figure 12b), whereas at 0 °C the discharging lifetime remains unchanged until an illumination intensity of 4 suns is reached. Both measurements demonstrate that that light-activated meta-stable trap state formation can be eliminated by altering their formation rate by lowering the temperature from 25 °C to 0 °C, which manifests as no degradation of PCE over time under constant 1-sun illumination.

Supplementary Note 5. Details on the photoluminescence and optical absorption results

Absolute absorption of a single large-area grain

The absolute absorption coefficient α (Supplementary Figure 13a) and absorbance $A(\omega)$ (Supplementary Figure 13b) were obtained from reflection and transmission microscopy measurements of the perovskite thin film at the single-crystal-grain level using two independent approaches details by Cesaria *et al.*[52] and Denton *et al.*[53] (details will be reported elsewhere). The method by Cesaria *et al.* uses the following relations for deriving the absorption properties:

$$A(\omega) = T_{\mathrm{S}}(\omega) + R_{\mathrm{S}}(\omega) - T(\omega) - R(\omega) \qquad \text{(Supplementary equation 3)}$$

$$\alpha(\omega) = \frac{1}{d}\left[\ln\left(\frac{1 - R(\omega)}{T(\omega)}\right) - \ln\left(\frac{1 - R_{\mathrm{S}}(\omega)}{T_{\mathrm{S}}(\omega)}\right)\right], \qquad \text{(Supplementary equation 4)}$$

where (R, T) stand for the overall reflection/transmission of the sample, and (R_{S}, T_{S}) are the reflection/transmission of the glass substrate. For the method by Denton *et al.*, R and T are modelled by Fresnel's equations for a two-layered system and assuming close-to-normal incident excitation light; this is justified since we used relatively low numerical aperture objective (50X, NA=0.45), and the substrate layer can be approximated by a semi-infinite layer with optical constants derived from our measurements of R_{S} and T_{S}. Typical thin film thicknesses d range from 280 to 320 nm in our samples.

Time-resolved photoluminescence

Experimental results

The time-correlated single photon counting histogram of the PL for the measurements presented in the main text (Fig. 2b) are sketched in Supplementary Figure 14. As explained in the main text, we considered two types of experiments both monitoring the light-excitation-power-density dependence of the time-resolved PL response of a single large-area perovskite grain:

(i) 'Continuous light soaking', the thin film is continuously illuminated and the power is varied from low to high light excitation power density and back to low power density.

(ii) 'Recovered system in dark', the sample is illuminated only during the measurement time and we waited for the system to recover in dark in between each data point of the power density cycle.

Recombination kinetics

The dynamics of carriers in crystal grain perovskite at room temperature were described by 1st order rate equations, after initial fast relaxation (~few ps) of free carriers to the band-edge unresolved in our measurements. In this picture, variations of the photo-generated excess carrier density *u(t)* at the band-edge are well described by:[25,54,55]

$$\dot{u} = G - \gamma_{\mathrm{b}} u^2 - \gamma_t u. \qquad \text{(Supplementary equation 5)}$$

The three terms on the right-hand side of (Supplementary equation 5) correspond to, respectively, the initial photo-excitation generation rate density of carrier (where the density of electrons is equivalent to the density of holes, calculated from the absorbance and the absorption coefficient α), the bimolecular radiation recombination (or spontaneous emission of light by the recombination of a free electron and hole at the band-edge), and the non-

radiative trap-assisted recombination of free carriers. The measured photoluminescence intensity can be expressed as:

$$\mathrm{I_{PL}} \propto \gamma_b u^2 + \gamma_b u N_D \quad \text{(Supplementary equation 6)}$$

where N_D corresponds to the total doping density of the material ($N_D = N_{\mathrm{electron}} + N_{\mathrm{hole}}$). Detailed analysis of the carrier recombination dynamics is beyond the scope of this paper and should be reported in future works. We emphasise that the relevant information in this paper is the comparison of the carrier effective lifetime[56] and PL integrated intensity between experiments (i) and (ii).

Decay lifetime and photo-emission vs. excitation power density

Following the modelling of the recombination kinetics described above of the data reported in Supplementary Figure 14, we observe differences between experiments **(i)** and **(ii)**. The effective decay lifetime (Fig. 2b) and integrated PL intensity (Supplementary Figure 15) were significantly reduced with prolonged illumination (light soaking) or excitation with high photon power density indicating the increase of trap-assisted non-radiative recombination, in good agreement with previous reports [24,54,57,58]. Furthermore, in the case of experiment (i), both the PL effective lifetime and PL intensity exhibit different photo-generated carrier density dependence when sweeping the excitation power density from high power to low power after reaching the maximum excitation power density. These observations can be correlated to the light-activation of non-radiative recombination via the meta-stable trap states described in the main manuscript and changes in the free-carrier density (charging effect) as observed via C-V measurements in solar cells. These results demonstrate the persistence of accumulated charged trap-states meta-stable during the

power decrease phase of the cycle in experiment (i), and testify of formation the light-activated meta-stable trap states.

Photo-bleaching effect and recovery mechanism

As mentioned in the previous sub-sections and the main text, the PL can be partially photo-bleached using high excitation power density. To better understand and correlate the mechanism of PL photo-bleaching to the photocurrent degradation observed in solar cells, we investigated the change of the power density threshold at which the photo-bleaching occurs for two temperatures (Supplementary Figure 16). We underline here that for this experiment we constantly illuminate the perovskite thin film at different power density (light soaking) and monitor the PL response at regular time intervals by temporarily lowering the power density to its lowest (where no degradation was observed). In this way, we were able to follow the evolution of the photoluminescence under the same conditions for all experiments, corresponding also to the PL monitoring conditions used for studying the recovery mechanism (Fig. 3b) in which case the sample is rested in the dark and the PL is probed at low power density at regular time intervals. At room temperature (25 °C), the power density photo-bleaching threshold is observed when illuminating the thin film with 20-30 $\mu J/cm^2$ corresponding to a clear quenching of the PL amplitude monitored at low power density. By heating the sample to 47 °C (which could better reflect the temperature of solar cells under standard operating conditions), the photo-bleaching threshold is lowered to few $\mu J/cm^2$. Therefore, considering the broadband excitation provided by 1-Sun light and the internal temperature increase of solar cells, we believe that PL spectroscopy (monochromatic light at 640 nm) is able to probe the same degradation mechanism. The strong temperature dependence observed here is in good agreement with the recovery dynamics and the model proposed in the main text.

Supplementary Note 6. Additional discussion on the origin of photo-degradation/self-healing

Ion migration

No observable XRD and PL changes

As illustrated in Supplementary Figure 3, we do not observe any structural change in XRD before and after illumination (i.e. no additional peak nor significant change in peak amplitudes). As suggested by previous reports[13,17], monitoring the photoemission of the materials over time and for several light cycles is instructive and might show a signature of ion migration. To this end we monitored the photoluminescence (PL) of our perovskite grains at regular time intervals for many cycles of constant illumination of the device and darkness (see Supplementary Figure 19). It should be noted here that we are very close to the operating conditions of a solar cell device. The integrated PL, peak emission energy, and full-width-at-half-maximum (FWHM) are remarkably stable over multiple light/dark cycles, independent on whether the sample is kept under light or in the dark.

Moreover, our devices do not show hysteresis in dark and under illumination (Fig. 1b in MS and ref.[24]). Several recent reports have attributed hysteresis effect to the ion vacancy/ ion migration through defects[17–19,21,22]. The large grain size perovskite films used in our measurements have a negligible contribution from vacancies or defects. This is further verified by C-V measurement in Supplementary Figure 10 where the large grain device (steady state) shows a fully depleted behavior in the junction, indicating there is no excess ion or ion vacancy in our system.

Therefore, we think our system has an inherently different behavior compared to the perovskite systems reported in the literature[13,17,21]. The absence of these standard signatures

of ion migration (changes in XRD and PL spectra or intensity) in the large grain-size perovskite thin-films also suggests that ion migration, if at all, is a negligible effect.

Impact on open circuit voltage

For a significant change in device performance, ions migrate from the bulk (excess ions) to the contact as suggested in the previous studies[20,21,59]. This should significantly alter the open circuit voltage as suggested in ref.[20] which shows switching behavior on V_{OC}. However, in our large grain-size perovskite PV devices, upon exposure to light, the V_{OC} reaches its maximum value in a few minutes and then remains stable as illustrated by the red curve in Supplementary Figure 20. In sharp contrast to this behavior observed for PV devices with large grains, similar measurements on PV devices with small grains (achieved by post-annealing method[1]) show that the V_{OC} reaches a steady state only after 30 minutes of light soaking, consistent with previous reports[23,60]. Such behavior has been attributed to ion migration in solid-state films with the presence of an external electric field. Moreover, Supplementary Figure 20 shows that a fit to the curve for small grain devices indeed follows the migration model proposed in ionic transporting materials[61,62]. For small grain devices, the short time scale can be fitted by a power law $\sim t^{1/2}$ while the longer term can be fitted into single exponential $\sim Exp(t/\tau)$. For large grain devices, this process is much shorter, indicating that ionic vacancies do not play a dominant role in large grain device as compared to small grain devices. A detailed comparison is beyond the scope of this work, and will be described in a future manuscript.

Moreover, the initial increase in V_{OC} with light soaking for the large grain devices presented in this manuscript occurs only when the device is illuminated the first time. After this, the voltage remains stable at its peak value even if it is sitting in the dark for tens of hours over several cycles of light ON and OFF as illustrated in Supplementary Figure 20b and Fig. 3b

in main text. However, for the small grain devices, the initial slow increase over several minutes in the V_{OC} is observed every time the device is illuminated. The peak value of V_{OC} also begins to slowly decay when sitting in the dark, starts to increase again upon shining light as illustrated by Supplementary Figure 20 and Supplementary Figure 21. This repeatable increase in light and decrease in dark for small grain devices, are consistent with previous reports[23,60]. It is clear that the different behavior with constant light excitation between large and small grain PV devices suggests that different mechanisms dominate during device operation under constant illumination.

Field dependence

According to recent reports, ion migration is significantly affected by applied external field[17,18,20,21]. Therefore, we examine our photocurrent degradation process under external field. Specifically we applied a forward bias to the photovoltaic device in the dark and monitor the photocurrent and open circuit voltage change as shown in Fig. 3c (main text). The results indicate that photocurrent does not degrade over time by biasing the device in the dark. On the contrary, the open circuit voltage increases slightly (± 0.05 mV) when a forward bias is applied in the dark. The voltage returns to its original value after removing the bias. This might be a consequence of ion migration. However, the miniscule change in V_{OC} of ± 0.05 mV again suggests that ion migration is negligible and is not the dominant mechanism that causes photocurrent degradation.

Hysteresis test for devices before and after photo-degradation

The device hysteresis effect is tested for device reaches a steady state and after degraded by 1 sun illumination for 2 hours. According to literature report[18,19], the ionic migration contributes to the hysteresis effect by fast *J-V* scans which will lead to a “S-shape” curve at

-1 V reverse bias. We therefore tested our hysteresis free device (Supplementary Figure 18, upper panel) for 2 hours constant illumination till the photocurrent degrades to 80% of its original value, and resulted hysteresis test are illustrated in Supplementary Figure 18 (lower panels), we do not observe hysteresis curves for different scan direction and scan rates. Therefore, we can conclude the constant illumination does not trigger ion migration induced *J-V* hysteresis and thus rule out any major contribution from ion migration.

Proposed mechanism

Dielectric constant change measured by capacitance

The relative dielectric constant change in Fig. 2d in MS is obtained by measuring the junction capacitance spectrum when applying an AC field (20 mV) as shown in Supplementary Figure 17. All the measurements are taken under dark. The capacitance can be expressed as

$$C = \varepsilon_0 \varepsilon_r \frac{A}{d} \qquad \text{(Supplementary equation 7)}$$

where A is area, *d* is device thickness, and ε_0 is vacuum permittivity.

The dielectric constant change in MS is calculated by subtracting the dielectric constant of steady state device (ε_i) from that of the charged device (same device after 2 hour light illumination) (ε_c) or it's recovered device (sitting in dark for few hours) (ε_r) and normalized by ε_i :

$$\Delta\varepsilon = \frac{\varepsilon_{c(r)} - \varepsilon_i}{\varepsilon_i} \qquad \text{(Supplementary equation 8)}$$

Raman scattering

Gottesman *et al.* recently proposed that Raman spectroscopy can be used to probe directly the effect of light on the libration modes of MA[33]. Supplementary Figure 22 sketches

preliminary Raman experiments conducted on our crystalline large-grain perovskite thin films. The pristine grain corresponds to the fresh sample illuminated at the detection limit of our experiment for very low excitation power, while the red curve is the Raman spectrum after light-induced degradation of the sample.

In both cases, we observe the main Raman peak of the perovskite around 110 cm^{-1},[33,63–65] and the lack of apparent Raman mode around 215 cm^{-1}, suggesting no significant degradation of the sample during the time of the measurement[66]. Comparing the two spectra, pristine and after light-induced degradation, we observe a change in the amplitude of the libration modes of MA (region 135-210 cm^{-1}) consistent with the recent report of Gottesman *et al.*[33]. On the other hand, the modes associated with the other elements of perovskite do change their relative intensity. This suggests that light (constant illumination over time or at high excitation fluence) affects directly the libration of MA and their Raman signature. An increase of Raman scattering intensity can be related to a strong slowing down of the MA motion, which entails a modification of the Raman scattering selection rules. Raman scattering observed in hybrid perovskites at high temperatures is thus related to distortions from the ideal structure.

Supplementary Note 7. Theoretical modeling of spatial localization of charge carrier wave function

Our study attributes the photo-degradation phenomena in perovskite solar cell devices to the light-activated meta-stable trap states of atomistic origin (polarons). Namely, we assume that there are two distinct types of charge states: free charges and polarons. The former have delocalized wave functions, low effective mass and are very mobile with diffusion length exceeding micron in the high quality crystalline perovskites. The second type of

charge carrier is a heavy polaron being a spatially localized electron or hole with a large effective mass and, therefore, very short diffusion length as illustrated on Supplementary Figure 23a,b. These carriers correspond to dressed quasiparticules, the dressing stemming in the non-polar case from the volumetric strain and interactions with neighboring MA ($CH_3NH_3^+$) cations that rotate so as to adiabatically follow the charge moving through the medium.

Symmetry analysis

To unravel the nature of light induced meta-stable trap states, symmetry consideration yields primary information about possible coupling between charge carriers and vibrational/rotational degrees of freedom. Starting with the parent cubic lattice, we analyze collective vibrations (phonons) and molecular reorientations (pseudo-spins) separately. Both of the most common notations for irreducible representations (IR) will be indicated: first that of Altmann[67] whereas that of Millers and Love[68] will be given in parenthesis. Lattice phonons involve atoms from the inorganic octahedra and the center of mass of the molecular cation. Therefore, molecular vibrations can be decomposed into two parts: translation of the center of mass and reorientation of the C-N axis when the cation is MA=$CH_3NH_3^+$.

Electron-phonon coupling occurs if the IR of a given phonon is contained in the product of IR of electronic states. For electronic states at the R-point of the Brillouin zone (BZ), the IR are A1g ($R1^+$) for the valence band (VB) and a triply degenerated T1u ($R4^-$) for the conduction band (CB). Taking into account spin-orbit coupling (SOC)[69], the IR become E1/2g for the VB, E1/2u for the bottom of the CB and F3/2u for the other states arising from spin-orbit split-off.[70] The product of these IR leads to: A1g ($\Gamma1^+$) for the VB and A1g+Eg+T1g+T2g ($\Gamma1^+$+$\Gamma3^+$+$\Gamma4^+$+$\Gamma5^+$) for the CB, without SOC. With SOC, it yields

A1g+T1g ($\Gamma1^+$+$\Gamma4^+$) for the VB as well as for the bottom of the CB and A1g+A2g+Eg+2T1g+2T2g ($\Gamma1^+$+$\Gamma2^+$+$\Gamma3^+$+2$\Gamma4^+$+2$\Gamma5^+$) for the other spin-orbit split-off CB states. Noteworthy, SOC is necessary to show that the top of VB and the bottom of CB have the same type of electron phonon coupling symmetry selection rules.

The discussion on the coupling mechanisms first focuses on the possible interactions between charge carriers and non-polar excitations (deformation potential theory). Taking the following atomic sites where the organic cation reduces to its molecular center of mass (M): Pb(0,0,0), M(1/2,1/2,1/2), 3X (1/2,0,0) (0,1/2,0) and (0,0,1/2), zone center optical phonons decompose along the 3T1u+T2u (3$\Gamma4^-$+$\Gamma5^-$) [70]. Thus optical phonons cannot directly couple to carrier transport close to the band gap. Deformation potential theory is at the heart of understanding the interaction of acoustic phonons and electrons.[71] Within this framework, the effect on the electronic band structure of an acoustic phonon is equivalent to that produced by a slowly varying strain in the direction of the phonon. Thus, when tacking SOC into account, electron-phonon coupling is allowed only for volumetric strain for which the IR is A1g ($\Gamma1^+$), both for VB and CB.[69]

Next, we consider molecular orientational degrees of freedom. To explain the order disorder character of the phase transitions from the cubic phase, Onoda et al.[72] introduced three possible scenarios (A, B and C) of molecular orientations for $CH_3NH_3PbX_3$. Model A, B and C correspond to thermally activated molecular tumbling occurring between the 6, 12 and 8 equivalent orientations of the C-N axis along direction [100], [110] and [111], respectively. Inclusion of the rotation of hydrogen atoms around the C-N axis adds further degeneracies (4, 2, and 3 for models A, B and C respectively). For each case, we define a pseudo-spin vector related to the distribution of reorientation probabilities between the equivalent positions. In a given cubic cell, one of the equivalent positions has an occupation

number of one whereas the others are vanishing. For instance, a general pseudo-spin of model C reads:

$$\left| \begin{matrix} \boldsymbol{N}_1 \\ \boldsymbol{N}_2 \\ \boldsymbol{N}_3 \\ \boldsymbol{N}_4 \\ \boldsymbol{N}_5 \\ \boldsymbol{N}_6 \\ \boldsymbol{N}_7 \\ \boldsymbol{N}_8 \end{matrix} \right.$$

where $\boldsymbol{N}_i$ is the occupation number of one of the eight [±1, ±1, ±1] orientations of the C-N axis in the crystal. Decomposition of the pseudo-spins IR at the Γ-point of the BZ yields:

Model A: A1g+Eg+T1u ($\Gamma 1^+ + \Gamma 3^+ + \Gamma 4^-$) ;

Model B: A1g+Eg+T2g+T1u+T2u ($\Gamma 1^+ + \Gamma 3^+ + \Gamma 5^+ + \Gamma 4^- + \Gamma 5^-$) ;

Model C: A1g+T2g+A2u+T1u ($\Gamma 1^+ + \Gamma 5^+ + \Gamma 2^- + \Gamma 4^-$).

This clearly shows that at the Γ-point of the BZ, the totally symmetric IR A1g is contained in all three decompositions. Therefore the electronic states can couple to any of the molecular arrangements out of the three models defining possible tumbling of the organic cations. Noteworthy, the totally symmetric $\Gamma 1^+$ IR corresponds to equal occupation probabilities for each orientation.

In summary, this symmetry analysis shows that free carriers can only undergo non-polar coupling through deformation potentials with acoustic phonons related to short-range interactions, local volumetric strain and to specific symmetric configurations of the molecular cations. The influence of local lattice deformations will be tested in the next part using DFT computations. Finally, let’s discuss the polar coupling mechanisms. The fact that low-frequency polar coupling to acoustic phonons, namely piezoelectric like mechanism[71], is symmetry forbidden in the Pm-3m cubic lattice, in addition to the scarcity of the

deformation potential mechanisms in relation with SOC[69], is most probably at the origin of good carrier transport and very large diffusion lengths[73] in these hybrid perovskites. The polar coupling to optical phonons, namely Fröhlich mechanism, is usually related to long-range interactions, the splitting between longitudinal and transverse phonon modes (LO-TO splitting) due a triply and polar degenerate IR (like $\Gamma 4^{-}$ for hybrid perovskites) as well as increments in the dielectric constant. This interaction is expected to occur in the hybrid perovskites at much higher frequencies (optical phonons energies in hybrid perovskites are on the order of 12meV i.e. 100cm^{-1}) than the interaction with low frequency acoustic phonons (which undergo energy dispersion down to 0 meV) or molecular pseudospins (typical relaxation times at 300 K amount to 5 ps i.e. 0.12 meV.[74] A polar coupling mechanism to pseudospins, connected to the triply degenerate and polar IR $\Gamma 4^{-}$ in the decompositions of the pseudo-spins IR, is thus expected to appear also at low frequency and yield additional contributions for the formation of polarons.

Effect of MA rotation and volumetric strain from DFT simulations

We start our DFT modeling using the bulk structure for the low temperature orthorhombic phase of MAPbI3.[75] In fact, it is well known that at room temperature (RT), the MA cations undergo dynamical disorder related to incompatible site symmetry with the RT space group and ad-hoc attempt to locate the MA in the RT unit cell introduces artificial long range dipole-dipole interactions. For this unperturbed structure, valence and conduction states reveal the well-established characteristics (Supplementary Figure 23c,d): an upper VB formed from the p orbitals of Iodine (in addition to s orbitals of Pb), and a lower CB formed from the unoccupied Pb p orbitals. Based on the above mentioned symmetry analysis, suitable models can be constructed to illustrate the formation of a polaron, first for specific

symmetric configurations of the organic MA cation and subsequently related to local volumetric strain.

To this end, we consider a supercell of 192 atoms. Consistently with electrostatic interactions, the cationic MA's are rotated towards an electron (hole) residing at one of the Pb (I) atoms, mimicking the creation of a 'heavy' charge carrier or polaron.[76] The nearest neighbor MA cations are first manually rotated so that the dipoles were either facing towards the chosen Pb atom (away from I). Then, the system is allowed to optimize for several optimization steps to reduce strain while retaining direction of MA dipoles. Supplementary Figure 23 (e,f) and (g,h) shows the changes on VB (HOMO) and CB (LUMO) states, respectively, induced upon rotating the nearest 8 (12) MA dipoles towards (away) from Pb (I). In the case where the nearest 8 MA cations are rotated towards a Pb cation the charge density of the HOMO remains highly delocalized while that of the LUMO becomes localized on that Pb atom (Supplementary Figure 23 (e,f)). This is indicative of the creation of a polaron for an excited electron in the conduction band minimum. When the nearest 12 MA ions are rotated away from a given I anion, the charge density of the HOMO becomes highly localized, illustrating the formation of a polaron for an excited hole in the valence band minimum, while the LUMO remains highly delocalized (Supplementary Figure 23 (g,h)). In both cases observed separation of charges is going to reduce radiative recombination between mobile and localized carriers.

Notably, there is a significant reduction of the band gap caused by orientation of the MA dipoles (Supplementary Figure 24). Regardless of whether or not SOC is included, the band gap in the system with rotated MA molecules is smaller by 0.1 to 0.2 eV compared to that of the unperturbed structure.

In order to model the effect of volumetric strain on the charge density, the experimental structure was first fully relaxed (forces < 0.025 eV/Å) allowing both atomic positions and the cell parameters to change. Then, considering a supercell of 192 atoms, a single Pb atom was replaced with either a Bi (+3 cation) or a Tl (+1 cation) atom and the new structure was optimized by just allowing the atomic positions to move. It is important to note that atoms surrounding the defect will move both because of the loss of charge balance as well as a change in atomic size. The changes in bond length, in pm, from the atoms immediately surrounding the defect atom are shown in Supplementary Table 1. In many ways these results are expected, as when the Bi atom is present, there is an additional positive charge that will cause the equatorial Iodine atoms to move closer, and the positively charge N atom to move farther way. It should be noted that the apical Iodine atoms actually move away from the Bi atom. This non-symmetric volumetric strain is likely a bias related to the use of an orthorhombic supercell. Tl, being only a +1 cation, will not attract the surrounding Iodine atoms as strongly and they will move farther way, while the MA atoms will want to be closer. The most probable reason for the much larger reorientation in the case of Bi substitution is the fact that Bi is much smaller than the similarly sized Tl and Pb.

Once the modified systems were optimized the charge densities were calculated. Supplementary Figure 25a,b shows a schematic of what volumetric strain may look like. Supplementary Figure 25c shows the localized charge density of the HOMO when Tl replaces a Pb atom. This is expected as Tl is a weaker cation than Pb. Supplementary Figure 25d shows the localized charge density of the LUMO when Bi replaces a Pb atom.

As further evidence of the change in charge density that volumetric strain can cause, the atomic coordinates that were obtained after optimization of the system containing a Bi atom were used to calculate the charge density of a system with all Pb atoms. The localized

HOMO is shown in Supplementary Figure 26. Here charge balance has been restored by reintroducing the original Pb atom, and only relative atomic displacement is causing the observed localization.

Density functional theory computation details

All theoretical computations presented are performed using the Vienna ab initio simulation package (VASP)[44,45] based on density functional theory (DFT) with the all-electron projected augmented wave (PAW)[45] method using the PBE exchange correlation functional.[40] The distributed PAW potentials have been generated by G. Kress following the recipes discussed in Kresse et al.[45]. Electron-ion interactions are described using with a kinetic energy cut-off of 525 eV. In order to make sure that our energy cutoff was high enough we conducted calculations using a 900 eV cutoff. Doing this only shifted the absolute energies by a couple tenths of meV, and the observed localization is still recovered. Valence states included the Pb 5d, 6s and 6p states, I 5s and 5p states, C 2s and 2p states, N 2s and 2p states, and the H1s state. We perform Brillouin-zone integrations using Monkhorst-Pack grids of special points with (4x4x4) meshes for the calculation of the structural and electronic properties. All calculations were done with and without spin-orbit coupling (SOC), and the overall conclusions of the results remained unaffected. Dispersion interactions can play a very important role in determining structural properties[78]. As such several test cases with the inclusion of van der Waals corrections to the DFT functional proposed by Dion et al.[46] and implemented into the VASP software[47,48] were performed. While in this case there were negligible changes in the band gaps and orbital charge densities, future investigations will focus on how dispersive forces play a role in the dynamics of the system.

Supplementary References

49. Kimerling, L. C. Influence of deep traps on the measurement of free-carrier distributions in semiconductors by junction capacitance techniques. *J. Appl. Phys.* **45,** 1839–1845 (1974).
50. Hegedus, S. S. & Shafarman, W. N. Thin-film solar cells: device measurements and analysis. *Prog. Photovolt. Res. Appl.* **12,** 155–176 (2004).
51. Decock, K. *et al.* Defect distributions in thin film solar cells deduced from admittance measurements under different bias voltages. *J. Appl. Phys.* **110,** 063722 (2011).
52. Cesaria, M., Caricato, A. P. & Martino, M. Realistic absorption coefficient of ultrathin films. *J. Opt.* **14,** 105701 (2012).
53. Denton, R. E., Campbell, R. D. & Tomlin, S. G. The determination of the optical constants of thin films from measurements of reflectance and transmittance at normal incidence. *J. Phys. Appl. Phys.* **5,** 852 (1972).
54. Lettieri, S., Capello, V., Santamaria, L. & Maddalena, P. On quantitative analysis of interband recombination dynamics: Theory and application to bulk ZnO. *Appl. Phys. Lett.* **103,** 241910 (2013).
55. Yamada, Y., Nakamura, T., Endo, M., Wakamiya, A. & Kanemitsu, Y. Photocarrier Recombination Dynamics in Perovskite CH3NH3PbI3 for Solar Cell Applications. *J. Am. Chem. Soc.* **136,** 11610–11613 (2014).
56. Berberan-Santos, M. N., Bodunov, E. N. & Valeur, B. Mathematical functions for the analysis of luminescence decays with underlying distributions 1. Kohlrausch decay function (stretched exponential). *Chem. Phys.* **315,** 171–182 (2005).
57. Manser, J. S. & Kamat, P. V. Band filling with free charge carriers in organometal halide perovskites. *Nat. Photonics* **8,** 737–743 (2014).
58. Stranks, S. D. *et al.* Recombination Kinetics in Organic-Inorganic Perovskites: Excitons, Free Charge, and Subgap States. *Phys. Rev. Appl.* **2,** 034007 (2014).
59. Zhao, Y. *et al.* Anomalously large interface charge in polarity-switchable photovoltaic devices: an indication of mobile ions in organic–inorganic halide perovskites. *Energy Environ. Sci.* **8,** 1256–1260 (2015).
60. Stranks, S. D. *et al.* Recombination Kinetics in Organic-Inorganic Perovskites: Excitons, Free Charge, and Subgap States. *Phys. Rev. Appl.* **2,** 034007 (2014).
61. Maier, J. *Physical Chemistry of Ionic Materials: Ions and Electrons in Solids*. (Wiley, 2004).
62. *Solid State Electrochemistry*. (Cambridge University Press, 1997).
63. Quarti, C. *et al.* The Raman Spectrum of the CH3NH3PbI3 Hybrid Perovskite: Interplay of Theory and Experiment. *J. Phys. Chem. Lett.* **5,** 279–284 (2014).
64. Grancini, G. *et al.* The Impact of the Crystallization Processes on the Structural and Optical Properties of Hybrid Perovskite Films for Photovoltaics. *J. Phys. Chem. Lett.* **5,** 3836–3842 (2014).

65. Park, B. *et al.* Resonance Raman and Excitation Energy Dependent Charge Transfer Mechanism in Halide-Substituted Hybrid Perovskite Solar Cells. *ACS Nano* **9,** 2088–2101 (2015).
66. Ledinský, M. *et al.* Raman Spectroscopy of Organic–Inorganic Halide Perovskites. *J. Phys. Chem. Lett.* **6,** 401–406 (2015).
67. Altmann, S. L. & Herzig, P. *Point-Group Theory Tables*. (Clarendon Press, Oxford, 1994).
68. S.C., M. & Love, W. F. *Tables of Irreducible Representations of Space Groups and Co-representations of Magnetic Space Groups*. (Pruett, 1967).
69. Even, J., Pedesseau, L., Jancu, J. M. & Katan, C. Importance of Spin − Orbit Coupling in Hybrid Organic/Inorganic Perovskites for Photovoltaic Applications. *J. Phys. Chem. Lett.* **4,** 2999–3005 (2013).
70. Even, J., Pedesseau, L. & Katan, C. Analysis of Multivalley and Multibandgap Absorption and Enhancement of Free Carriers Related to Exciton Screening in Hybrid Perovskites. *J. Phys. Chem. C* **118,** 11566 (2014).
71. Yu, P. Y. & Cardona, M. *Fundamentals of Semiconductors*. (third edition Springer, 2005).
72. Onoda-Yamamuro, N., Matsuo, T. & Suga, H. Calorimetric and IR spectroscopic studies of phase transitions in methylammonium trihalogenoplumbates (II). *J. Phys. Chem. Solids* **51,** 1383–1395 (1990).
73. Dong, Q. *et al.* Electron-hole diffusion lengths > 175 mm in solution-grown CH 3 NH 3 PbI 3 single crystals. *Science* **347,** 967 (2015).
74. Poglitsch, A. & Weber, D. Dynamic disorder in methylammoniumtrihalogenoplumbates (II) observed by millimeter - wave spectroscopy Dynamic disorder in methylammoniumtrihalogenoplumbates (II) observed by millimeter-wave spectroscopy. *J. Chem. Phys.* **87,** 6373 (1987).
75. Baikie, T. *et al.* Synthesis and crystal chemistry of the hybrid perovskite (CH3NH3)PbI3 for solid-state sensitised solar cell applications. *J. Mater. Chem. A* **1,** 5628 (2013).
76. Zhang, W., Govorov, A. O. & Ulloa, S. E. Polarons with a twist. 1–4 (2002). doi:10.1103/PhysRevB.66.060303
77. Perdew, J., Burke, K. & Ernzerhof, M. Generalized Gradient Approximation Made Simple. *Phys. Rev. Lett.* **77,** 3865–3868 (1996).
78. Egger, D. A. & Kronik, L. Role of Dispersive Interactions in Determining Structural Properties of Organic–Inorganic Halide Perovskites: Insights from First-Principles Calculations. *J. Phys. Chem. Lett.* **5,** 2728–2733 (2014).
